# Individual-Level Exposure to Light at Night and Sleep Health: A Comparison between Real-Time Mobility-Based Measurements and Indoor Residence-Based Measurements

**DOI:** 10.1021/acs.est.5c08270

**Published:** 2025-10-26

**Authors:** Yuhan Cui, Mei-Po Kwan, Yang Liu

**Affiliations:** † Department of Geography and Resource Management, 26451 The Chinese University of Hong Kong, Shatin, Hong Kong SAR 999077, China; ‡ Institute of Space and Earth Information Science, The Chinese University of Hong Kong, Shatin, Hong Kong SAR 999077, China

**Keywords:** exposure measurements, mobility-based LAN, indoor LAN, sleep health, UGCoP

## Abstract

Light at night (LAN) disrupts human circadian rhythms and impairs sleep health. However, inconsistent findings across observational studies using indoor residence-based measurements (IRBM) and real-time mobility-based measurements (RMBM) raise methodological concerns. This study investigated IRBM and RMBM of LAN exposure and their associations with sleep health, examining sex as a potential moderator. Data from 484 individuals (aged 18–65) over 1,748 nights were collected in Hong Kong, incorporating objective (actigraphy) and subjective (sleep diary) measures. Concurrent LAN exposure was quantified via LUX29TK portable light meter (IRBM) and GENEActiv wrist-worn actigraph-embedded luxmeter (RMBM). Generalized linear mixed models adjusted for sociodemographic, temporal, geographic, physical activity, health status, and lifestyle covariates. Initial analyses revealed differences between IRBM and RMBM with inconsistent effects. For LAN across astronomical night (sunset to sunrise), covariate adjustment attenuated contextual discrepancies in IRBM models. Both methods demonstrated negative associations between LAN and objective sleep duration and efficiency, with no effects on subjective sleep or sex moderation. For LAN across biological night (sleep onset to wake), RMBM exhibited instrumental error, yielding divergent associations. These findings reveal how contextual and instrumental errors between IRBM and RMBM across analysis windows explain inconsistencies in prior LAN-sleep research, informing reliable exposure quantification in sleep studies.

## Graphical abstract

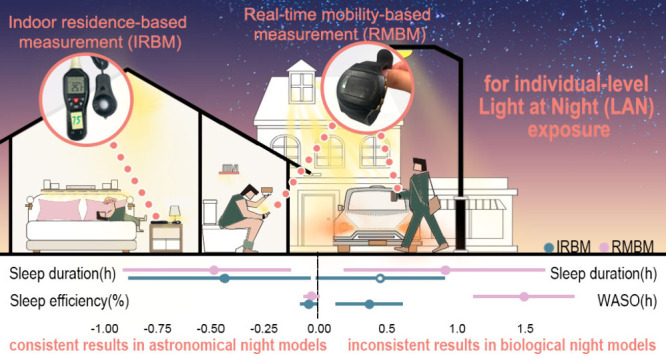

## 1 Introduction

Light at night (LAN), exacerbated by ongoing urbanization, represents a widespread environmental pollutant affecting over 80% of the global population.^1,2^ While offering conveniences like improved visibility, security, and extended activity hours, this pervasive exposure poses a significant public health burden.^3^ Research consistently demonstrates that urban populations, especially those in highly industrialized areas, experience shorter sleep durations and later sleep timing compared to their rural counterparts.^3^ For instance, town dwellers in the Brazilian Amazon were observed to have significantly shorter sleep durations, delayed sleep onset, and later wake-up times compared to rural ones.^4^

LAN impairs sleep through two distinct yet complementary physiological pathways. It fundamentally disrupts human circadian rhythms by desynchronizing the internal biological clock from the natural light-dark cycle and suppressing the nocturnal secretion of melatonin, while concurrently acting via a direct alerting pathway that acutely promotes wakefulness and fragments sleep.^3,5,6^ This dual physiological disturbance compromises sleep health, a multidimensional construct encompassing duration, efficiency/continuity, timing, alertness/sleepiness, and satisfaction/quality.^7^ The degradation of sleep health, in turn, serves as a critical pathway mediating LAN’s broader effects on cognitive impairment, negative moods, metabolic diseases, and increased cancer risk.^8–12^

Experimental studies have linked personal LAN exposure to multiple sleep health dimensions, including decreased sleep duration, fragmented sleep continuity, delayed sleep timing, declined next-morning alertness and reduced sleep satisfaction.^13–17^ However, these studies are often conducted in well-controlled laboratory settings or interventional studies of specific populations (e.g., nightshift workers), thereby limiting the broader applicability of the findings to the general population under everyday conditions.^18,19^

Observational studies often employ two primary approaches to measure LAN exposure: radiometric and photometric.^20^ Radiometric measurements, derived from remote sensing data, provide estimates of outdoor LAN exposure in urban environments, mainly capturing ambient-derived light pollution.^21^ These measurements, however, have limited ability to capture individual-level exposure,^22^ particularly from nonambient indoor light sources, leading to low correlations with actual indoor LAN exposure.^22–24^ In contrast, photometric measurements, using illuminance devices like photometers or actigraphy embedded with a light sensor, offer a more accurate assessment of individual-level light exposure, including those generated indoors by personal behaviors, which are hard for remote sensing images to capture.^20^

While photometric measurements have been employed to account for light exposure from indoor sources, and to mitigate potential measurement bias with the radiometric approaches, another frequently overlooked source of exposure measurement error remains: the context in which researchers try to capture an individual’s LAN exposure.^25–27^ This contextual variation, specifically the methodological distinction between indoor residence-based measurements (IRBM) and real-time mobility-based measurements (RMBM), may contribute to past inconsistencies in research findings,^18,23,24^ as residence-based studies found associations between bedroom LAN and poorer sleep health,^28,29^ whereas mobility-based studies showed no such correlation, or associations that were conditional on factors like sex and race/ethnicity.^30–32^ For example, personal LAN exposure was found to be associated with shorter sleep duration among females but not males,^32^ a finding potentially explained by sex differences in circadian physiology and light sensitivity.^33,34^

Further, a growing body of environmental health research highlights the critical role of human mobility in accurately assessing environmental exposures, such as air pollution and, relevantly, LAN.^25–27^ The IRBM approach, by using stationary sensors to measure ambient LAN exposure within a fixed home environment, fails to capture the true spatiotemporal contexts of exposure due to time-activity errors, a challenge articulated as the uncertain geographic context problem (UGCoP).^35,36^ In contrast, the RMBM approach, which employs wearable sensors to capture a more ecologically valid record of personal light exposure as an individual moves through various environments, is likely to mitigate these contextual errors. However, to what extent the UGCoP influences the effects of personal LAN exposure on sleep health has not been empirically examined.^37^ Further, existing research shows that evening light exposure — defined as LAN exposure in the presleep hours — impacts subsequent sleep by suppressing melatonin and delaying circadian rhythms, a key mobility-dependent factor that supports the practical implications of mobility-based measurement in this field.^38–41^

Beyond the UGCoP, however, a second challenge arises from instrumental error, particularly measurement artifacts that can bias readings even when location is held constant. A crucial unresolved question is whether IRBM and RMBM devices provide comparable data even under ideal ‘apples-to-apples’ real-life conditions, such as during the in-bed sleep period. While RMBM captures a more ecologically valid record of personal light exposure, they may overrepresent behavioral patterns and be sensitive to body movements, potentially failing to detect light accurately. Examining contextual error from mobility and instrumental error from sensor artifacts is essential for valid inference in LAN research, yet they have not been empirically examined.

To tackle the existing inconsistency in the association between personal LAN exposure and sleep health, and to explore the role of context-related variations between residence-based and mobility-based measurements, this study utilizes cross-sectional survey data from Hong Kong. As one of the world’s most densely populated cities (over 6,756 people/km^2^ by 2022 ^42^), Hong Kong presents an ideal research setting, with night sky brightness 82 times higher than international dark-sky standards and urban areas 15 times brighter than rural areas.^43^ The absence of comprehensive light pollution legislation,^44^ combined with a high prevalence of poor sleep health among residents (over 10% of individuals aged 15 or above),^45^ makes Hong Kong particularly suitable for this research. This study aims to address the following research questions: 1) How does contextual error influence the relationship between LAN and sleep? We compare IRBM and RMBM of individual-level LAN exposure over the astronomical night and assess how statistical adjustment for night-level and individual-level covariates, guided by a directed acyclic graph (DAG) (Figure S1), mitigates their differences. 2) How does instrumental error manifest when contextual error is minimized? We conduct the comparison during the biological night (actigraphy-derived sleep period) to isolate instrumental error and assess how statistical adjustment influences their differences. 3) How do these sources of error alter the LAN-sleep associations, and does sex moderate these relationships?

## 2 Materials and Methods

### 2.1 Study Design

From November 2021 to April 2023, 800 adults aged 18–65 were recruited from four neighborhoods in Hong Kong (see Text S1): Sha Tin (ST), Kwun Tong (KT), Kwai Tsing (KQ), and the Central and Western districts (CW) (Figure S2). After the data screening process, data from 1748 observation nights for 484 participants were obtained. The exclusion criteria of participants (N) and data records (n) were (see Text S2 for the reasons): if the lux meter or lux meter embedded in the actigraph devices was detected to have the missing values (N = 239), or if the actigraph device was detected to have less than 10 h of wearing time during the night (N = 16), or if any of their self-reported data were missing (N = 55), or if they had fewer than two consecutive days of data (N = 6), or if the recording sleep night was the first night by the actigraph device (n = 443), or if valid hours in a calendar day were less than 16 h for detection of physical activity (n = 22). The study protocol was reviewed and approved by the Survey and Behavioral Research Ethics Committee of the Chinese University of Hong Kong (approval numbers: SBRE-19–123 and SBRE­(R)-21–005). Informed consent was obtained from all participants before data were collected from them.

To ensure that the data cleaning process did not introduce selection bias, we compared the distributions of key demographic variables between the included participants and the excluded ones. No statistically significant differences were observed (all *p* > 0.05), indicating that the cleaned data set remains representative of the original sample (Table S1). Summary characteristics of the participants are provided in Table S2–3 (discussed in Section 3.1 below).

### 2.2 Measures

#### 2.2.1 Individual-Level LAN Exposure

RMBM was measured by GENEActiv, a wrist-worn, waterproof actigraph device (Activinsights Ltd., Kimbolton, UK). This device is equipped with a silicon photodiode sensor, featuring a wavelength range of 400–1100 nm, a measurement range of 0–3000 lx, and an accuracy of ± 10% at 1000-lx calibration. The GENEActiv was set to record at 100 Hz and exported to 1-s epochs by GENEActiv Windows Software from Activinsights. Then the LAN exposure data of RMBM were down-sampled into 1-minute epochs.^46^ Since the GENEActiv was reported to underestimate illuminance compared to a laboratory photometer and had no manufacturer-provided calibration,^47^ the absolute illuminance data were corrected using the toolbox by Joyce et al.(2020).^46,48^ This processing pipeline is consistent with the methodology detailed by Joyce et al.(2020).^46^

IRBM was measured at 1 min intervals using a portable light meter (LUX29TK; Tekcoplus Ltd., Hong Kong, China). This meter complied with the International Commission on Illumination (CIE) standard and featured a measurement range of 0–200,000 lx, an accuracy of ± 4% below 10,000 lx, and a sampling time of 0.5 s. The light sensor was positioned near the head of the bed and directed toward the ceiling.

The light meter and actigraph devices were synchronized to ensure their internal clocks were aligned. A review of the literature revealed no consensus on a standard LAN definition (Table S4). Therefore, to explicitly dissect sources of measurement error, we operationalized LAN exposure in two distinct ways. First, astronomical night was calculated as the average light intensity from local sunset to sunrise,^49^ providing a population-level probe that accounted for interindividual variability in circadian phase sensitivity.^40^ This window served to capture the combined effect of real-world contextual error (due to mobility) and instrumental error. Second, biological night was calculated as the average light intensity from actigraphy-derived sleep onset to wake-up time. This window created a quasi-experimental condition that minimized contextual error, allowing for the isolation of instrumental error.

#### 2.2.2 Sleep Parameters

Subjectively measured sleep parameters were collected from participants using standardized sleep diaries. After each night, participants were required to report their sleep time, wake-up time, daytime nap start and end times, number of nighttime awakenings, and sleep quality. The response options for sleep quality were “Poor,” “Fair,” and “Good.”.^50,51^ The reported number of nighttime awakenings was subsequently reclassified into “0”, “1”, “2”, and “≥3” categories for analysis.^52^ Self-reported nighttime awakenings and sleep quality were used as subjectively measured sleep parameters.

Objectively measured sleep parameters were derived from GENEActiv with the help of the corresponding R-package GGIR (ver 3.1–0) (Hees et al., 2024). GENEActiv is a triaxial accelerometer that records acceleration magnitude when people carry out their daily activities. Based on the accelerometer-derived data, GGIR detects the arm angle and its relation to the horizontal plane. Fewer changes in arm angle were classified as sleep.^53,54^ In addition, sleep diaries were directly entered into the algorithm to calculate time in bed. The following objectively measured sleep parameters were examined: (1) sleep duration (SD), time spent between sleep onset time and wake-up time; (2) sleep efficiency (SE), sleep duration divided by time in bed derived from sleep diaries; (3) wake after sleep onset (WASO), duration of wakefulness between sleep onset time and wake-up time; (4) sleep onset latency (SOL), the difference between sleep onset and time in bed. Full details can be found in Code S1 in Supporting Information.

#### 2.2.3 Covariates

Sociodemographic information, health status, lifestyle behaviors, season and geographic contexts were observed to be related to sleep outcomes in past studies and may provide individual-level contextual information about LAN exposure.^32,33,45,55–65^ We therefore collected the data via our survey questionnaires guided by the directed acyclic graph (DAG) (Figure S1).^66^ Temporal contexts and sleep-related contexts were obtained from the sleep diaries. It is noteworthy that the use of sleep medication, which was reported by 28 person-nights in the sleep diaries, failed to meet one or more of our inclusion criteria stated in Section 2.1 and was thus removed during our data screening process. Daytime physical activity was calculated from GENEActiv data using the GGIR package.^67^ Full details can be seen in Text S3 in Supporting Information.

### 2.3 Statistical Analysis

LAN exposure data were first log10-transformed before all the follow-up measures because raw light exposure data have a right-skewed distribution and circadian responses to light exhibit a log–linear correlation.^68^ The sleep onset latency variable with a right-skewed distribution was also naturally log-transformed for analyses.^69^ Descriptive statistics characterized the variable distributions. The agreement between IRBM and RMBM was assessed using Bland-Altman analysis corrected for repeated measures.^70^ Mann–Whitney U tests and Kruskal–Wallis rank sum tests were used to compare how the difference between IRBM and RMBM varies by temporal and geographic contexts. Generalized variance inflation factors (GVIF) were used to test multicollinearity between all the independent variables, and the GVIF across all models was lower than 2, which shows no multicollinearity between the independent variables.^71^ Separate generalized linear mixed models (GLMMs) were conducted for LAN exposure by IRBM and RMBM symmetrically (see Text S4). We used stratified analysis, which means increasing levels of variables to adjust the models (Figure 1). Wald tests were conducted to examine whether the coefficients of LAN exposure measured by IRBM and RMBM were significantly different across residence-based models and mobility-based models.^72^

**Figure 1:**
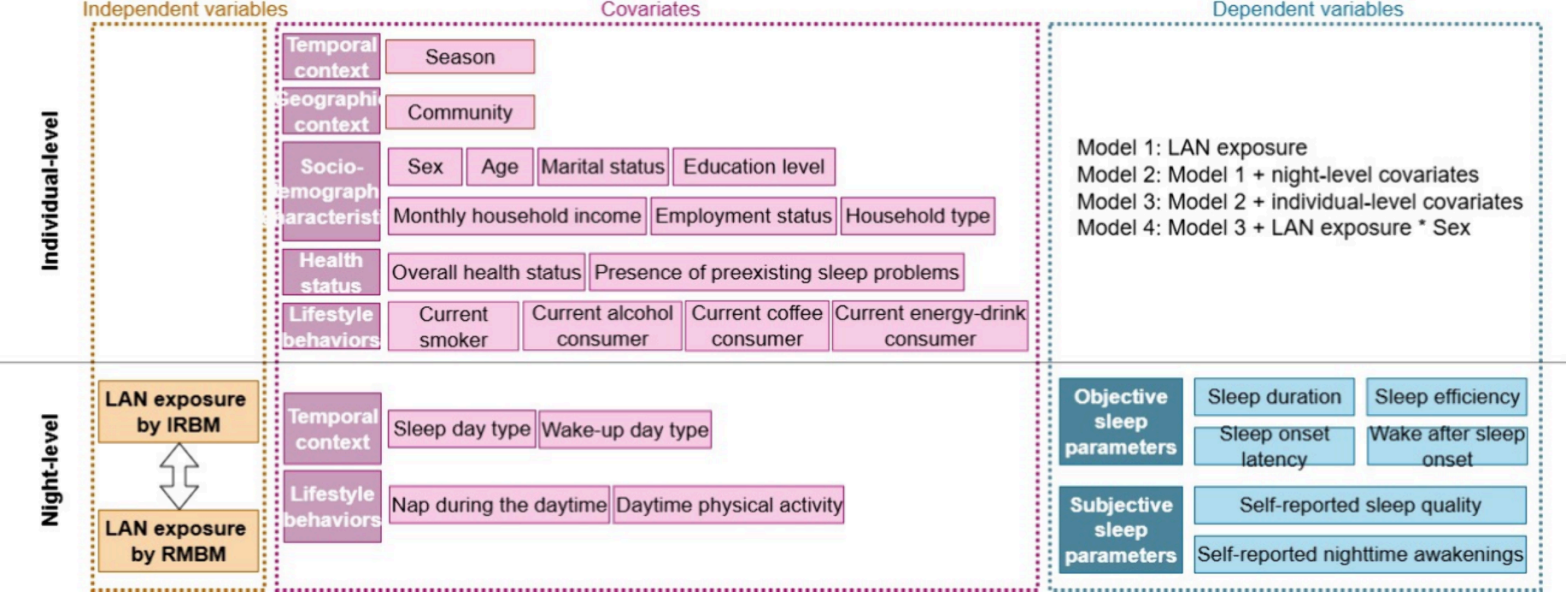
Study design for multilevel models. Model 1: only LAN exposure was entered as the fixed effect; Model 2: Model 1 + adjusted for night-level variables: nap during the daytime, sleep day type (weekday or weekend day), wake-up day type (weekday or weekend day), and daytime physical activity; Model 3: Model 2 + adjusted for individual-level variables: community, season, sex, age, marital status, education level, monthly household income, employment status, household type, overall health status, presence of preexisting sleep problems, current smoker, current alcohol consumer, current coffee consumer, current energy-drink consumer; Model 4: Model 3 + an interaction term between personal LAN exposure and sex, to assess potential effect moderation for all the sleep outcomes.

To assess the influence of covariates on the magnitude and precision of the estimated effect of LAN exposure on sleep parameters, we adapted the backward change-in-estimate concept, which was initially designed for model selection, to perform a post hoc covariate impact analysis.^73^ Because we observed that the magnitude and statistical significance of the LAN-sleep associations, as well as the consistency between IRBM and RMBM findings, changed considerably with increasing model adjustment. We developed two data-driven metrics: the change-in-estimate (CIE) and the change-in-standard error (CISE), to study the relative impact of specific covariates on the final adjusted LAN-sleep associations. This impact analysis involved the following steps: First, we used the fully adjusted model as the baseline model (Model 3). Second, we constructed a series of reduced models by individually omitting each potential confounder, one at a time, from Model 3. Third, for each reduced model, we calculated the two metrics. The CIE, calculated as

$$CIE=\frac{\left(\beta_{full}-\beta_{reduced}\right)}{\beta_{full}}\times 100\%$$

where β_*full*_ is the LAN exposure coefficient from the fully adjusted model, and β_*reduced*_ is the LAN exposure coefficient from the model with the targeted confounder removed. The CIE for each potential confounder was used to assess its contribution to the estimation of the LAN exposure effect. Similarly, the CISE, calculated as

$$CISE=\frac{\left({SE}_{full}-{SE}_{reduced}\right)}{{SE}_{full}}\times 100\%$$

where *SE*_*full*_ is the standard error of the LAN exposure coefficient from the fully adjusted model, and *SE*_*reduced*_ is the standard error after removing the targeted confounder. The larger negative value of CISE indicated the better relative contribution of a confounder to the precision of LAN exposure estimates in the final model. We summarized the CIE, and CISE for each confounder.

All statistical analyses were conducted in R version 4.4.0, with the help of the *lme4* package for the GLMMs with the continuous response variables, the *ordinal* package for the GLMMs with the ordinal response variables, as well as *texreg* and *ggpubr* for result visualization.^74–77^

## 3 Results

### 3.1 Descriptive Results

The final sample did not include any missing data, and the sample size was 484 people with 1748 nights (Table S2–3). Each participant had an average of 3.61 nights, with weekdays accounting for the majority of sleep day type (60.98%) and wake-up day type (71.05%). For the study population, Sha Tin district had the highest proportion of participants (37.19%), followed by Kwun Tong and Kwai Tsing Districts, and Central and Western District had the least (12.6%). Moreover, most participants were female (67.77%), middle-aged (61.16%), middle-income level (40.91%), and full-time workers (59.92%).

### 3.2 Comparison of LAN Exposure by IRBM and RMBM across Astronomical Night

Figure 2A displays the Bland-Altman plot assessing the agreement between the log-transformed IRBM and RMBM data. The plot revealed a mean bias of −0.16 log lux, and 95% limits of agreement (LoA) ranged from −1.73 to 1.42 log lux. The 95% confidence interval (CI) for the mean bias was consistently negative, suggesting systematically higher values of LAN exposure captured by RMBM compared to IRBM. The near-zero regression coefficient (β: −0.05, with 24.1% of the bootstrap-derived (N = 1000 resamples) p-values for the slope were less than 0.05) indicated a negligible proportional bias. The wide range of LoA suggested considerable random errors between the two measurement approaches.

**Figure 2:**
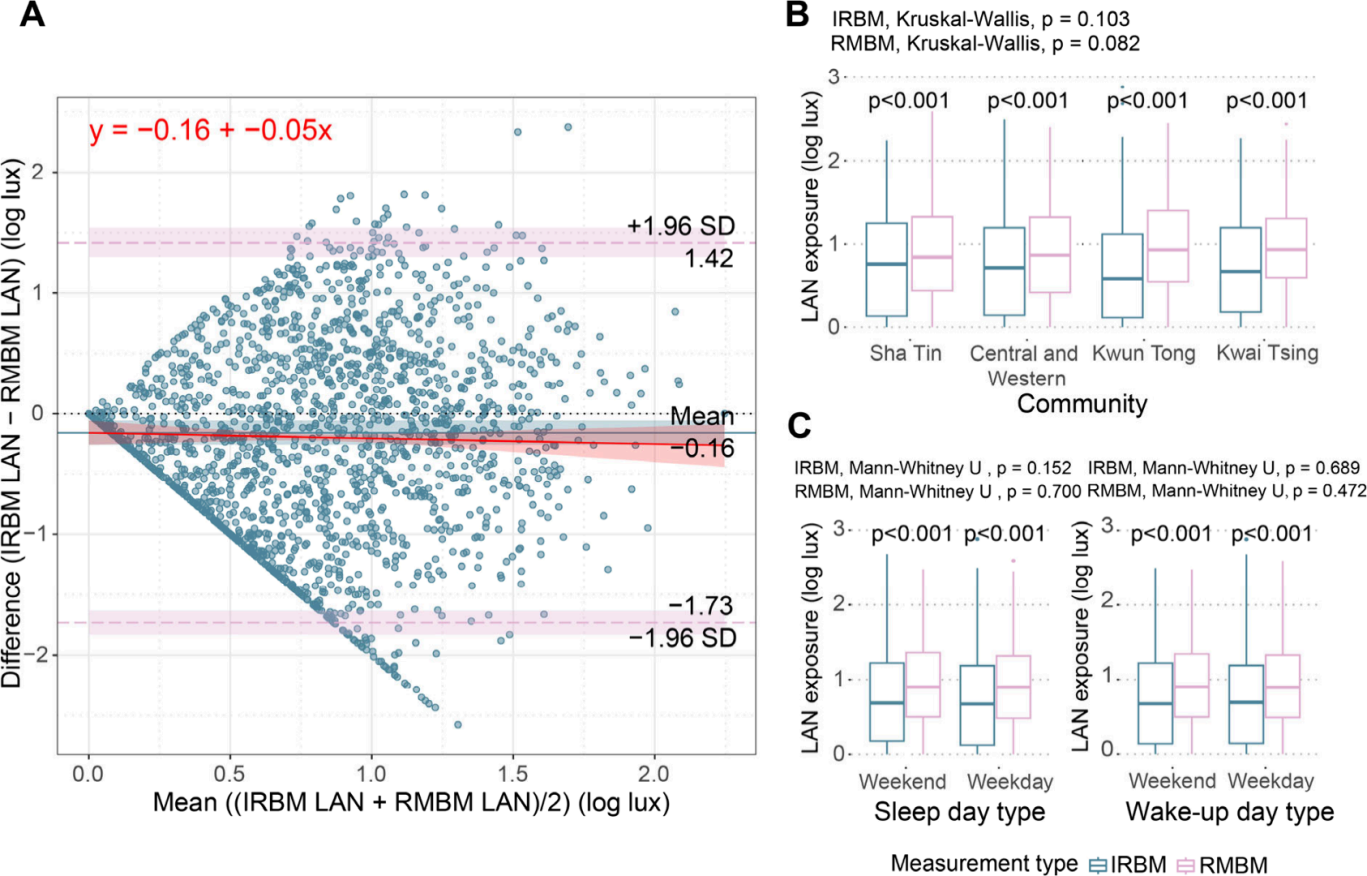
Discrepancies between IRBM and RMBM for LAN exposure across astronomical night. (A) Bland-Altman plot comparing IRBM and RMBM. A regression line was fitted to assess proportional bias. (B) Geographic disparities in LAN exposure by IRBM and RMBM. (C) Temporal disparities in LAN exposure by IRBM and RMBM. In (B) and (C), green and pink represent IRBM and RMBM values, respectively.

Figures 2B and 2C show the difference between LAN exposure by IRBM and RMBM across geographic and temporal contexts. LAN exposure was significantly different between measurement methods (*p* < 0.001), and LAN exposure measured by RMBM (0.93 ± 0.56 log lux) was greater than IRBM (0.73 ± 0.59 log lux) under all circumstances. However, personal LAN exposure did not have disparities across geographic and temporal contexts for either measure (*p* > 0.05). Full details can be seen in Tables S5 and S6 in Supporting Information.

Figure 3 reports the models of associations between LAN exposure and sleep parameters according to IRBM and RMBM, with increasing levels of adjustment (Table S7). In Model 1, which only included the LAN exposure variable, LAN exposure measured by RMBM has a significant negative association with sleep duration (β: −0.55; 95% CI: −0.94, −0.16) and sleep efficiency (β: −0.04; 95% CI: −0.07, −0.00), while IRBM models did not show this association. However, the increase in LAN exposure measured by IRBM would lead to lower log odds of being above a particular self-reported nighttime awakening level (OR: 0.39; 95% CI: 0.18, 0.85), which was not observed in RMBM models. In Model 2, which adjusted for night-level variables, LAN exposure measured by IRBM turned to a significant negative association with sleep duration (β: −0.44; 95% CI: −0.86, −0.02). Other associations did not change. Then, in Model 3, which was the fully adjusted model, LAN exposure has a significant negative association with sleep duration and sleep efficiency for both measurement types (sleep duration: IRBM: β: −0.43; 95% CI: −0.86, −0.01; RMBM: β: −0.48; 95% CI: −0.87, −0.09; sleep efficiency: IRBM: β: −0.04; 95% CI: −0.08, −0.01; RMBM: β: −0.03; 95% CI: −0.07, −0.00), while none of the significant relationships was shown in other models. Additionally, according to Wald tests, comparable coefficients or odds ratios of LAN exposure by IRBM and RMBM did not significantly differ from one another (*p* > 0.05, Table S7). Full details about the stratified analysis can be found in Table S8–S19.

**Figure 3:**
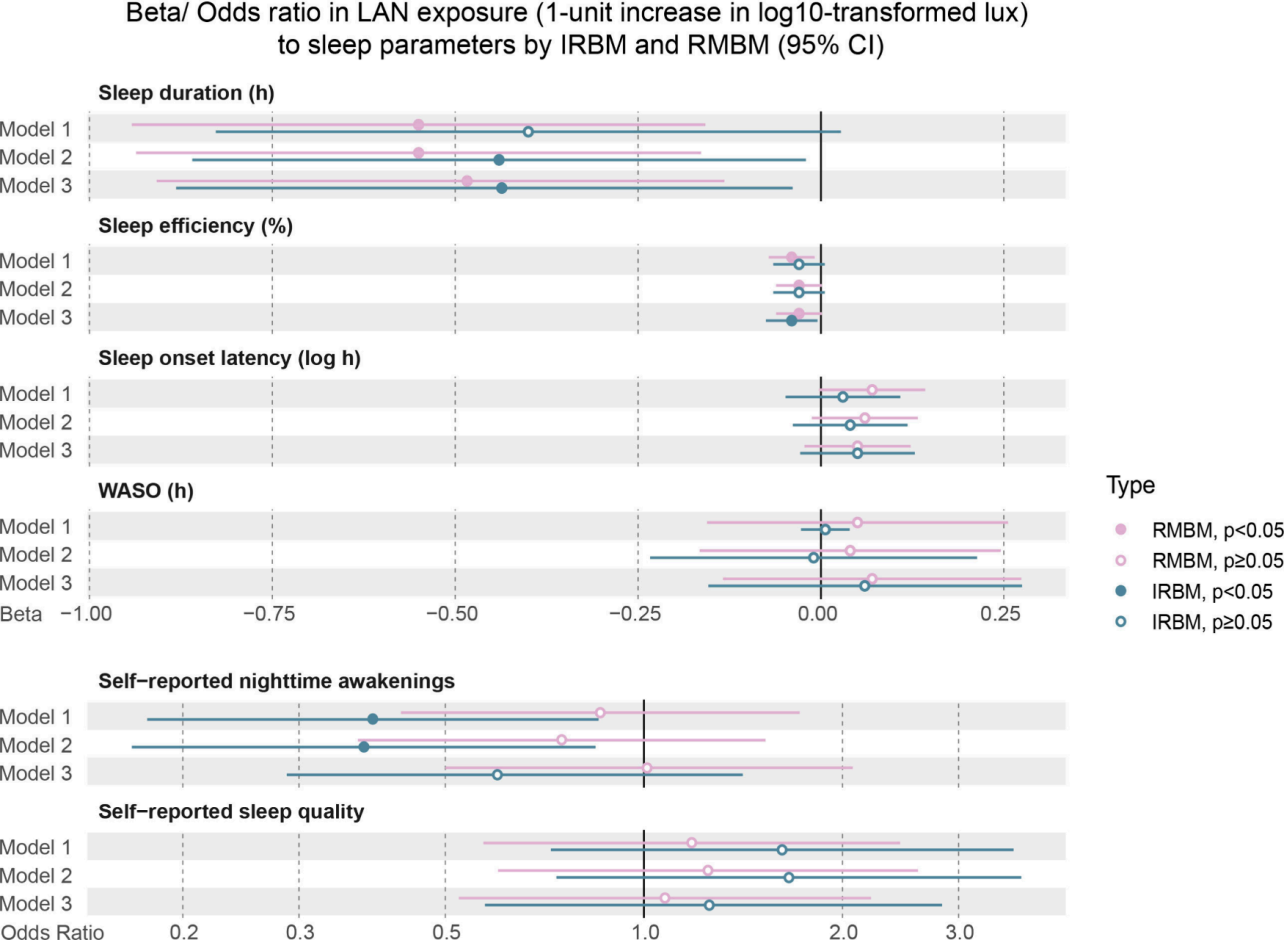
Comparisons of beta/odds ratio in LAN exposure across astronomical night (1-unit increase in log10-transformed lux) to sleep parameters by IRBM and RMBM (95% CI). Model 1: only LAN exposure was entered as the fixed effect; Model 2: Model 1 + adjusted for night-level variables: nap during the daytime, sleep day type (weekday or weekend day), wake-up day type (weekday or weekend day), and daytime physical activity; Model 3: Model 2 + adjusted for individual-level variables: community, season, sex, age, marital status, education level, monthly household income, employment status, household type, overall health status, presence of preexisting sleep problems, current smoker, current alcohol consumer, current coffee consumer, current energy-drink consumer. Estimates are presented as Beta coefficients for continuous outcomes and Odds Ratios for ordinal outcomes. Self-reported sleep quality (categorized as “Poor” [reference], “Fair”, “Good”). Self-reported nighttime awakenings (categorized as “0” [reference], “1”, “2”, “≥3”).

Figures 4A and 4B depict the percentage change-in-estimate (CIE) and change-in-standard error (CISE) for the LAN exposure coefficient in IRBM models, focusing on sleep duration and sleep efficiency – the only sleep outcomes significantly associated with LAN exposure after progressively adjusting the covariates. For sleep duration, individual-level confounders exhibiting CIE values exceeding 5% included monthly household income, age, and season. Among night-level confounders, daytime physical activity showed a CIE exceeding 3%. These results highlight the relative importance of these variables in confounding the LAN-sleep duration association. Notably, wake-up day type had the largest impact on estimate precision, with a CISE of −1.47%, suggesting its contribution to reducing the standard error of the LAN exposure estimate. Regarding sleep efficiency, monthly household income (individual-level) and sleep day type (night-level) also showed considerable confounding effects, with CIEs exceeding 5% and 3%, respectively. Sex exhibited the greatest improvement in precision, with a CISE of −0.64%, indicating a reduction in the standard error of the LAN estimate upon its inclusion. Comprehensive details are provided in Tables S20 and S21. To test whether the powerful confounders also act as effect modifiers, we conducted moderation analyses by introducing interaction terms between LAN exposure and both age and monthly household income. For sleep duration, a significant interaction was found between LAN exposure and age (*p* < 0.05), indicating that the negative effect of LAN on sleep duration was stronger in older participants. However, no significant interaction was found with age for sleep efficiency, nor with monthly household income for either outcome (Tables S8 and S10, Models 5–6).

**Figure 4:**
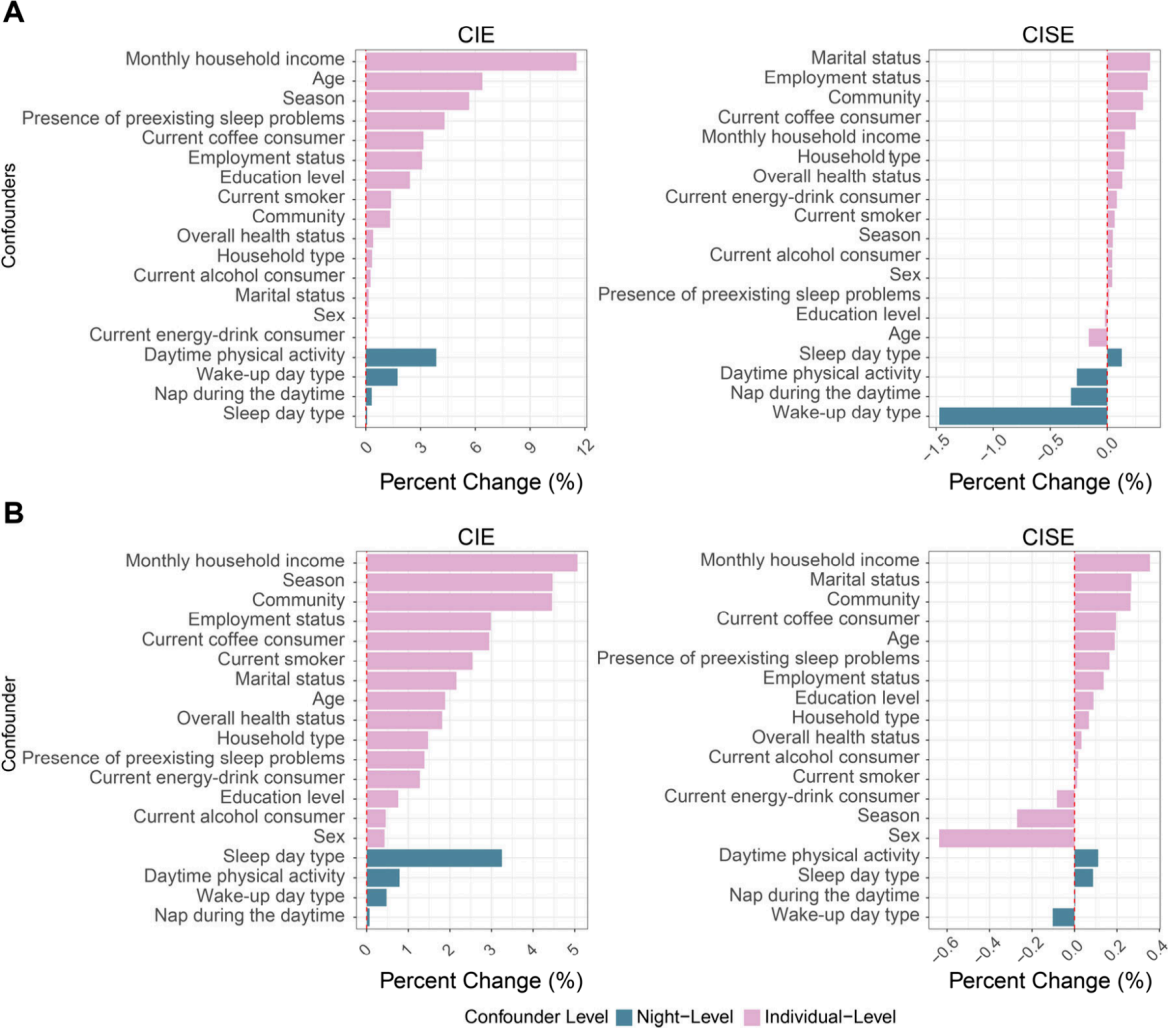
Impact of individual confounder removal on the LAN exposure coefficient (CIE and CISE) in IRBM models. (A) Results for sleep duration. (B) Results for sleep efficiency. Green indicates night-level confounders; pink indicates individual-level confounders.

Interaction terms between personal LAN exposure and sex were incorporated into all fully adjusted IRBM and RMBM models for all sleep outcomes analyzed. The absence of statistically significant interactions indicated that stratification by sex was not warranted. Detailed results are provided in Tables S8–S19 (Model 4) of the Supporting Information.

### 3.3 Comparison of LAN Exposure by IRBM and RMBM across Biological Night

Figure 5A displays the Bland-Altman plot comparing IRBM and RMBM. It revealed a mean bias of 0.01 log lux and 95% limits of agreement (LoA) ranged from −0.70 to 0.72 log lux. The 95% confidence interval (CI) for the mean bias was consistently positive, suggesting systematically higher values of LAN exposure captured by IRBM compared to RMBM. The regression coefficient was 0.59, which indicated a proportional bias, where the discrepancy between the devices increased with higher light intensity.

**Figure 5:**
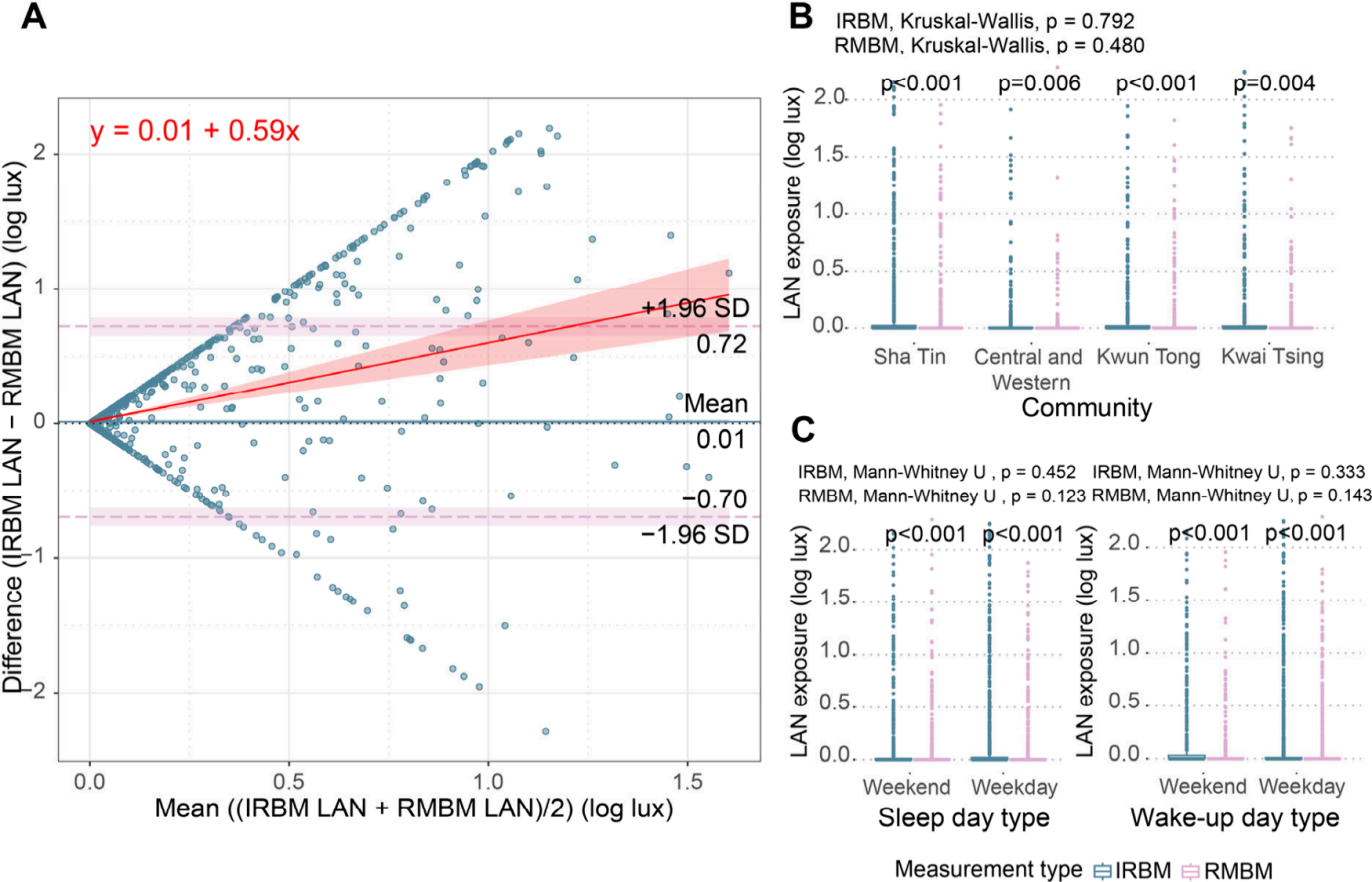
Discrepancies between IRBM and RMBM for LAN exposure across biological night. (A) Bland-Altman plot comparing IRBM and RMBM. A regression line was fitted to assess proportional bias. (B) Geographic disparities in LAN exposure by IRBM and RMBM. (C) Temporal disparities in LAN exposure by IRBM and RMBM. In (B) and (C), green and pink represent IRBM and RMBM values, respectively.

Figures 5B and 5C show the difference between LAN exposure by IRBM and RMBM across geographic and temporal contexts. LAN exposure was significantly different between measurement methods (*p* < 0.05), and LAN exposure measured by IRBM (0.14 ± 0.38 log lux) was greater than RMBM (0.07 ± 0.24 log lux) under all circumstances. However, personal LAN exposure did not have disparities across geographic and temporal contexts for either measure (*p* > 0.05). Full details can be seen in Tables S22 and S23 in Supporting Information.

Figure 6 reports the models of associations between LAN exposure and sleep parameters according to IRBM and RMBM, with increasing levels of adjustment (Table S24). Since biological night was defined by an objective sleep period, analyses for sleep efficiency and sleep onset latency were omitted. The distinct signals captured by each device revealed more nuanced associations with sleep outcomes: (a) Sleep Duration. The IRBM showed no significant main effect on sleep duration. In contrast, the RMBM showed a significant positive association (Model 3: β=0.91, p = 0.013). Importantly, the sex-interaction model for IRBM became significant (p = 0.017), indicating that higher ambient LAN during sleep was associated with shorter sleep duration for females but not males. This subtle relationship was not detected by the RMBM. (b) Wake After Sleep Onset (WASO). Both methods revealed a significant positive association between LAN and WASO. Notably, the RMBM produced a much larger effect estimate (β=1.48 vs β=0.38 for IRBM in Model 3, *p* < 0.001). (c) Self-Reported Nighttime Awakenings. While main effects were not significant, the sex-interaction models were. For both methods, higher LAN was associated with more self-reported awakenings in females compared to males. (d) Self-Reported Sleep Quality. The IRBM showed a significant interaction, with higher ambient LAN being associated with poorer self-reported sleep quality in females. This effect was not observed with the RMBM. Full details about the stratified analysis can be found in Table S25–S32.

**Figure 6:**
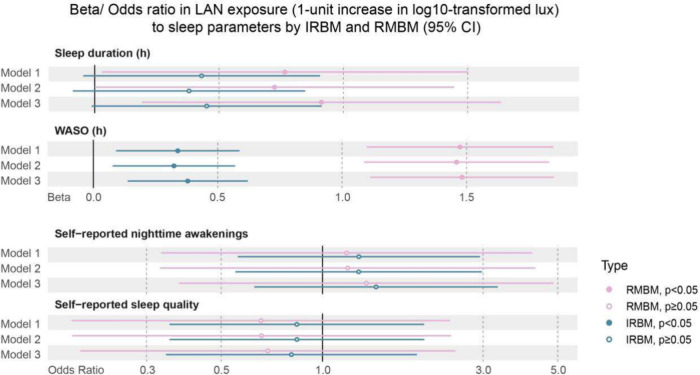
Comparisons of beta/odds ratio in LAN exposure across biological night (1-unit increase in log10-transformed lux) to sleep parameters by IRBM and RMBM (95% CI). Model 1: only LAN exposure was entered as the fixed effect; Model 2: Model 1 + adjusted for night-level variables: nap during the daytime, sleep day type (weekday or weekend day), wake-up day type (weekday or weekend day), and daytime physical activity; Model 3: Model 2 + adjusted for individual-level variables: community, season, sex, age, marital status, education level, monthly household income, employment status, household type, overall health status, presence of preexisting sleep problems, current smoker, current alcohol consumer, current coffee consumer, current energy-drink consumer. Estimates are presented as Beta coefficients for continuous outcomes and Odds Ratios for ordinal outcomes. Self-reported sleep quality (categorized as “Poor” [reference], “Fair”, “Good”). Self-reported nighttime awakenings (categorized as “0” [reference], “1”, “2”, “≥3”).

## 4 Discussion

LAN is a growing environmental pollutant associated with urbanization. However, the relationship between personal LAN exposure and sleep health in real-world settings remains unclear, raising questions about the potential influence of exposure measurement methodologies.^18^ Leveraging Hong Kong as a high-density urban case study, this research provides one of the first comprehensive examinations of LAN-sleep associations using both IRBM and RMBM concurrently. Despite differences in absolute LAN exposure values, our findings reveal largely consistent associations between personal LAN exposure and detrimental sleep effects across both measurement approaches. However, the nature of this link and the primary sources of measurement error differ critically depending on the exposure window. During astronomical night, IRBM is prone to contextual error by failing to account for individual mobility. Nonetheless, both methods identified consistent negative associations, likely reflecting shared circadian phase-shifting mechanisms. During biological night, RMBM becomes susceptible to instrumental error, creating measurement artifacts. In this context, IRBM models provided clearer signals, linking ambient bedroom light to reduced sleep health in females. This suggests that IRBM misses presleep behavioral exposures, but RMBM may be sensitive to in-bed or within-sleep behaviors. For example, IRBM may better measure ambient bedroom light, while RMBM may better measure LAN due to periods of wake (e.g., turning on a bathroom light). Therefore, measurement choice must align with specific research questions, weighing contextual versus instrumental error risks.

### 4.1 Systematic Difference between IRBM and RMBM

During astronomical night, RMBM consistently recorded higher LAN exposure than IRBM, irrespective of geographic or temporal contexts. While device sensitivity differences to various light spectra might contribute, including infrared light where the GENEActiv watch (RMBM) may exhibit sensitivity while typical photopic lux meters (IRBM)^47^ may not, individual mobility omission is the principal driver of the observed divergence. To our knowledge, this study is the first to explicitly compare residence-based and mobility-based measurements of personal LAN exposure. Nevertheless, our finding aligns with research examining other environmental exposures, such as green space, blue space, air pollution, and noise,^72,78–80^ where mobility-based assessments frequently record higher exposures than residence-based estimates. These discrepancies reflect diverse contextual information captured by each approach rather than inherent environmental characteristics.^81^ IRBM is prone to significant exposure misclassification arising from locational mismatches by recording at fixed residential locations regardless of participant presence. For instance, if a participant is away from their residence—such as engaging in nighttime outdoor activities (when bedroom lights are typically off) or simply being in another room of the house for extended periods—the IRBM continues to measure the unoccupied residential environment. This type of contextual error, similar to time-activity error in epidemiological literature, represents the dominant source of divergence between the two methods when using the behavior-inclusive time window.^82,83^

Conversely, during biological night, this relationship was inverted: IRBM values were consistently higher than RMBM, irrespective of geographic or temporal contexts. With locational differences minimized (participants in bed), main error sources shift from contextual to instrumental. Bland-Altman analysis revealed proportional bias, where RMBM underestimation worsened with increasing ambient light intensity, suggesting physical masking of wrist-worn sensors by bedding or body becomes the primary error driver.^32,84^

### 4.2 Consistent LAN-Sleep Associations in IRBM and RMBM Models

During astronomical night, both IRBM and RMBM identified consistent negative associations between individual-level LAN exposure and sleep duration/sleep efficiency, corroborating previous field studies.^28,29,32^ This consistency, despite RMBM recording significantly higher absolute exposures, suggests that with robust covariate adjustment, both methods successfully capture shared biological signals: circadian-disrupting effects of LAN exposure activating intrinsically photosensitive retinal ganglion cells (ipRGCs) to delay sleep-promoting physiology, especially during the late evening and early night.^5,6,40,85^ In this context, RMBM directly measures personal LAN exposure during evening activities, while IRBM acts as a strong proxy for household light habits. Extensive adjustments appear to isolate this biological mechanism from behavioral confounders, thereby unmasking the common biological pathway captured by both the IRBM and RMBM, despite the potential for instrumental and time-activity errors to attenuate effect estimates and increase standard errors.^86–88^ Similar findings have been reported in coexposure studies, where the presence of contextual error did not substantially alter overall conclusions.^72^ However, no significant main effects were found on sleep onset latency, WASO, or self-reported sleep parameters, nor sex-based moderation. These null findings align with some research^30,32^ but diverge from others,^69^ possibly due to inadequate confounding control in past studies, operational LAN definitions across astronomical night, obscuring nuanced timing and spectral effects,^41,55,85,89–91^ and inherent sensor limitations attenuating true association, such as the sensitivity to causally irrelevant LAN or the accuracy to physiologically meaningful variations in dim light conditions.^47^

During biological night, our findings uncovered more complex and method-dependent associations. Both methods revealed consistent positive associations between LAN exposure and WASO, corroborating previous field studies.^28,29^ However, IRBM revealed significant sex-specific interactions, where higher ambient bedroom LAN was detrimentally associated with sleep duration, self-reported nighttime awakenings, and self-reported sleep quality exclusively in females, aligning with literature on heightened female sensitivity.^32,92^ RMBM models failed to detect most relationships, identifying only significant sex interaction for self-reported nighttime awakenings with substantially larger effect estimates, pointing to instrumental error influence during sleep periods.^93^

### 4.3 Contextual Error between IRBM and RMBM during Astronomical Night

Progressive IRBM model adjustment revealed shifts in LAN-sleep associations as confounders were controlled. Key influential covariates include monthly household income, age, season, daytime physical activity, and sleep day type. Additionally, wake-up day type and sex contributed to explaining variance in sleep duration and sleep efficiency respectively, thereby impacting the standard errors of the LAN coefficients in the IRBM models.

For confounders that adjusted the LAN effect estimates in the IRBM models, variables captured inherent personal characteristics influencing sleep outcomes while interacting with spatiotemporal behavioral patterns, leading to a complex interplay with exposure measurements.^55^ Socio-demographic variables (monthly household income and age) linked to individual variations^29,56^ and mobility differences contribute to IRBM-RMBM discrepancies.^82,94^ Beyond this primary role as confounders, moderation analysis revealed that while monthly household income acted purely as a confounder, age also functioned as an effect modifier, with stronger adverse LAN effects on sleep duration in older adults.^29^ Temporal variables (season, sleep day type) affected sleep outcomes and LAN exposure through temperature, day length, and behavioral differences between weekdays/weekends.^55,57–59^ Lifestyle behavior variable (daytime physical activity) correlated with sleep duration, with variations based on age and sex,^60^ and reflected mobility patterns.^95^

For confounders that primarily affected the precision of the LAN effect estimates (i.e., their standard errors) in the IRBM models by explaining additional variance in sleep outcomes. Prior research supports the influence of wake-up day type (related to social jetlag) on sleep duration and the tendency for women to exhibit increased sleep efficiency compared to men.^96,97^

In contrast, RMBM models exhibited remarkable consistency across all adjustment levels, suggesting RMBM captures more comprehensive LAN exposure and is less susceptible to contextual factors. The intrinsic ability potentially reduces the need for extensive covariate adjustment. As confounders were added to IRBM models, coefficient estimates converged toward RMBM models, providing evidence that incorporating confounders corrected time-activity-related contextual error rather than introducing bias.^86^ Our finding challenges a previous study that questioned the utility of RMBM, reinforcing its validity for exposure assessment in this context.^72^

### 4.4 Instrumental Error between IRBM and RMBM during Biological Night

During biological night, RMBM became compromised by instrumental error, manifesting as paradoxical findings. First, our findings revealed positive associations between RMBM-measured LAN exposure and sleep duration, a result that contradicts the well-established biological mechanism of LAN-induced melatonin suppression and its sleep-disrupting effects. We therefore interpret this as a spurious association, likely attributable to GENEActiv’s infrared sensitivity capturing physiological sleep progression rather than ambient light.^47,98^ Second, RMBM may have been affected by physical masking, leading to chronic underestimation of true LAN exposure, which may explain why IRBM captured female-specific associations that RMBM did not.^93^ Taken together, these findings suggest that in some instances, RMBM may bias results toward the null. These differences in IRBM and RMBM may also help explain existing inconsistencies in the literature, where null findings could reflect measurement error rather than a true lack of effect.^32,99^

### 4.5 Limitations and Future Directions

This study contributes to the literature by highlighting how LAN exposure measurement methods impact sleep study outcomes and helps explain conflicting results. However, limitations exist. First, the cross-sectional design cannot exclude a reverse association whereby poor sleep leads to LAN exposure. While controlling baseline preexisting sleep problems mitigates this, reverse causality cannot be fully ruled out on a night-to-night basis. Especially for outcomes such as wake after sleep onset (WASO), while we observed positive LAN-WASO associations, the causal pathway may plausibly be reversed, as periods of wakefulness could prompt movement or rising from bed, thereby triggering light exposure events. More experimental interventions are needed in real-life settings to confirm the causal relationship.^100^ Second, comparison is constrained by inherent method limitations. The wrist-worn actigraph (RMBM) offers high ecological validity but suffers bidirectional measurement bias. It can underestimate exposure when the sensor is obscured by clothing or bedding,^18^ and the GENEActiv device can overestimate it due to its sensitivity to nonvisual infrared light.^47,101^ Conversely, the stationary luxmeter (IRBM) features a diffuser dome for robust measuring of ambient room illuminance but lacks contextual validity regarding mobility and biologically relevant exposure.^101^ This highlights fundamental trade-offs and underscores the need for novel sensors uniting precision with ecological validity. Third, exposure to other environmental factors (noise, air pollution, green space, blue space, or heavy metal), may confound LAN-sleep relationships.^100,102–104^ We excluded these variables to avoid introducing additional exposure errors.^72,105^ Future studies can examine this association more systematically. Fourth, we did not assess bedroom settings (blackout curtains, eye masks) serving as sleep hygiene proxies, which may slightly overestimate LAN-sleep associations, though extensive behavioral and health confounders likely mitigate most confounding.^106^ Future studies should include detailed bedroom environment measures.

## Supplementary Material



## Notes

Directed acyclic graph (DAG); study area in Hong Kong; recruitment process of participants; rationale for data exclusion criteria; the reason for selecting generalized linear mixed models; data collection method for covariates; comparison of included and excluded participants; characteristics of participants and night-level variables; definition of LAN exposure by IRBM and RMBM in the past literature; LAN exposure by IRBM and RMBM across geographic and temporal contexts; Beta/Odds ratio in LAN exposure to sleep parameters by IRBM and RMBM and results of Wald’s test; stratified analysis for sleep duration, sleep efficiency, sleep onset latency, WASO, self-reported nighttime awakenings, and self-reported sleep quality by IRBM and RMBM, respectively; impact of covariates removal on LAN-Sleep duration/Sleep efficiency association in IRBM models; settings when processing the data with GGIR; and results of astronomical night analysis and biological night analysis (PDF)
