# Supplementary material for: Individual-Level Exposure to Light at Night and Sleep Health: A Comparison between Real-Time Mobility-Based Measurements and Indoor Residence-Based Measurements
Source: Environ Sci Technol. 2025 Oct 26;59(43):23349–61. doi: 10.1021/acs.est.5c08270 (PMC12593368; doi:10.1021/acs.est.5c08270)
Supplement: Supplementary file 1 [file es5c08270_si_001.pdf]

Supporting Information for

Individual-level Exposure to Light at Night and Sleep Health: A Comparison between Real-time Mobility-based Measurements and Indoor Residence-based Measurements

Yuhan Cui [1, 2], Mei-Po Kwan [1, 2, *], and Yang Liu[1, 2]

[1] Department of Geography and Resource Management, the Chinese University of Hong Kong, Hong Kong Special Administrative Region of China

[2] Institute of Space and Earth Information Science, the Chinese University of Hong Kong, Hong Kong Special Administrative Region of China

*Corresponding author: Mei-Po Kwan mpk654@gmail.com

The file includes:

Figure S1 to S2

Text S1 to S4

Table S1 to S32

Code S1

References

Figure S1. Directed acyclic graph (DAG).

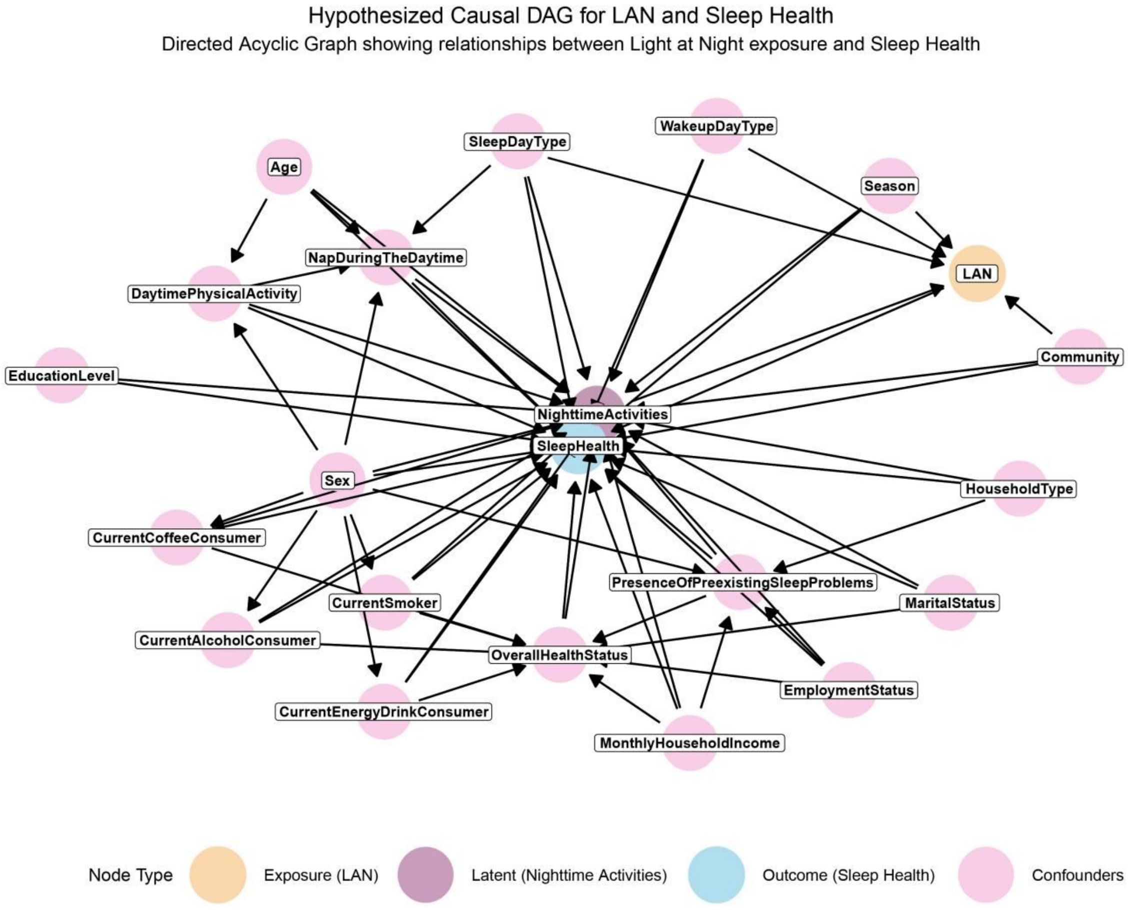

Figure S2. Study area in Hong Kong.

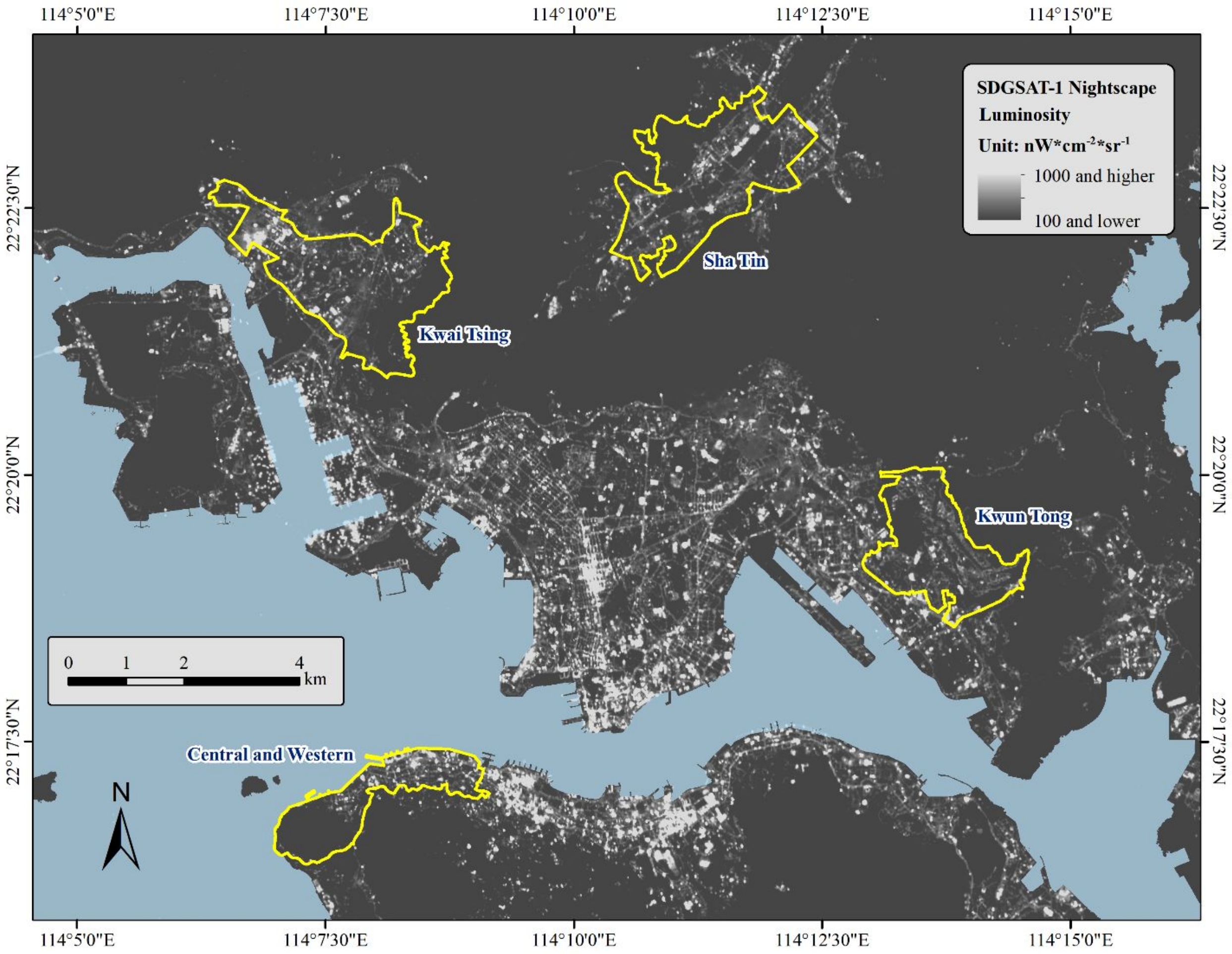

Text S1. Recruitment process of participants.

Participants were chosen by stratified random sampling according to residents' sex, age, marital status, and socioeconomic status in each neighborhood. Ethics approval was obtained from the Survey and Behavioral Research Ethics (SBRE) Committee of the Chinese University of Hong Kong (Reference No. SBRE-19-123 approved on January 8, 2020, and Reference No. SBRE(R)-21-005 approved on November 1, 2021). All participants provided their written informed consent before data was collected from them. After obtaining written informed consent, participants were requested to complete questionnaires containing sociodemographic information, health status, and lifestyle behaviors. Then, they were asked to wear an actigraph device on their non-dominant wrist, which was embedded with a light sensor, for real-time mobility-based measurements (RMBM) of LAN exposure. They were also asked to put a portable photometer in their bedroom, which was on the nightstand facing the ceiling, for indoor residence-based measurements (IRBM). Participants were required to be conscious of not covering the actigraph device. At the same time, they were requested to keep a sleep diary, which at least included one weekday and one weekend day.

Text S2. Rationale for data exclusion criteria.

For 'if the lux meter or lux meter embedded in the actigraph devices was detected to have the missing values (N=239)', we ensure that all analyzed nights have complete and directly comparable light exposure recordings from both measurement methods, thereby preventing bias or misinterpretation due to incomplete data streams; for 'if the actigraph device was detected to have less than 10 hours of wearing time during the night (N=16)' and 'if valid hours in a calendar day were less than 16 hours for detection of physical activity (n=22)', we set these thresholds based on the GGIR parameter 'includenightcrit=10' and 'includedaycrit = 16', to ensure a reliable assessment of sleep parameters and daily physical activity levels[1–3];for 'if any of their self-reported data was missing (N=55)', we maintain a complete dataset for all covariates included in the statistical models; for 'if they had fewer than two consecutive days of data (N=6)', we utilize a repeated-measures design which requires a minimum of two valid nights per participants for robust mixed-effects modeling; for 'if the recording sleep night was the first night by the actigraph device (n=443)' , we have three considerations for this: (a) As the study protocol initiated at 8 PM, the first night's light recording often did not capture the full nocturnal period from sunset, potentially leading to an

underestimation of total light exposure for that night. (b) The first night of wearing a monitoring device can lead to atypical sleep patterns due to participant adaptation. Excluding this night minimizes potential bias from these unrepresentative sleep data, a common practice in sleep research [4,5]. (c) Physical activity, a covariate, was assessed using a "wake-to-wake" window. The first recorded night would lack a complete preceding day's activity record under this definition.

Text S3. Data collection method for covariates.

Sociodemographic information, health status, lifestyle behaviors, season and geographic contexts were collected via our survey questionnaires. Sociodemographic characteristics include sex, age, marital status (single, married, others), education level (middle school or lower, bachelor's degree or certification, master's degree or higher), monthly household income (less than 20,000 HKD, HKD 20,000-39,000, and HKD 40,000 or above), employment status (full-time, part-time, freelance, student, unemployed), and household type (couple with child, couple without child, single parent, and others). Health status includes overall health status (poor or good) and the presence of preexisting sleep problems (yes or no). Lifestyle behaviors include current smoker, current alcohol consumer, current coffee consumer, and current energy-drink consumer, which are all dichotomous variables. The 'season' variable was categorized based on the month of participation. The seasons were defined as follows: Spring (March to May), Summer (June to August), Autumn (September to November), and Winter (December to February). Geographic contexts contain four communities (ST, KT, KQ, CW), which are the four communities in Hong Kong selected for this study. Temporal contexts and sleep-related contexts were obtained from the sleep diaries, including nap during the daytime (yes or no), sleep day type (weekday or weekend), and wake-up day type (weekday or weekend). Sleep day type and wake-up day type refer to the day on which the participant initiated sleep before and woke up after the sleep episode, which enables us to analyze sleep patterns across different types of days (weekday and weekend day) of the week. Daytime physical activity was calculated from GENEActiv data using the GGIR package [7]. Analyses were based on the wake-up to wake-up time window, during which period daytime physical activity summary statistics were derived. The thresholds for differentiating inactivity, light, moderate, and vigorous physical activity were 30, 100, and 400 counts/min, respectively. Then, according to the formula provided by the International Physical Activity Questionnaire (IPAQ) Reliability Study, metabolic equivalent of task (MET) minute values were generated based on a

scoring protocol (walking 3.3 METs, moderate 4 METs, and vigorous 8 METs), and then the MET-minutes scores were calculated to represent the daytime physical activity variable [8].

Text S4. The reason for selecting generalized linear mixed models (GLMMs).

Because each participant has more than two repeated nights for analysis and participants were recruited from four communities, the nested data structure suggested we use the generalized linear mixed models (GLMMs). We calculated the intraclass correlation coefficient (ICC) to decide the multilevel structure of our dataset. Regarding the continuous dependent variables, like sleep duration, sleep efficiency, wake after sleep onset, and sleep onset latency, we employed between-group variance to partition the total variance for ICC. For the ordinal dependent variables, like self-reported nighttime awakenings, and self-reported sleep quality, we used the between-group variance to partition $\pi^2/3$, which is the variance of the logistic distribution. All the ICCs at the community level were close to zero (range: 0.001 to 0.012), indicating negligible between-community variance. The ICCs at the individual level for sleep duration, sleep efficiency, wake after sleep onset, sleep onset latency, self-reported nighttime awakenings, and self-reported sleep quality were 0.29, 0.37, 0.35, 0.20, 0.59, and 0.56 respectively, so we employed two-level GLMMs with night-level variables and individual-level variables. For the continuous response variables, full maximum likelihood estimation was used. For ordinal response variables, adaptive Gauss-Hermite quadrature was used [6].

Table S1. Comparison of included (N=484) and excluded (N=316) participants.

Characteristic	Category	Included participants, n(%)	Excluded participants, n(%)	Total, n(%)	Test
Community	Sha Tin	180 (57.51%)	133(42.49%)	313(39.12%)	p value: 0.2838 (Pearson's Chi-squared test)
	Central and Western	61 (63.54%)	35 (36.46%)	96 (12.00%)	
	Kwun Tong	137 (65.24%)	73 (34.76%)	210 (26.25%)	
	Kwai Tsing	106 (58.56%)	75 (41.44%)	181 (22.62%)	
	Total	484 (60.50%)	316 (39.50%)	800 (100.00%)	
Season	Spring	85 (59.03%)	59 (40.97%)	144 (18.00%)	p value: 0.7444 (Pearson's Chi-squared test)
	Summer	31 (60.78%)	20 (39.22%)	51 (6.38%)	
	Autumn	123 (58.02%)	89 (41.98%)	212 (56.50%)	
	Winter	245 (62.34%)	148 (37.66%)	393 (49.12%)	
	Total	484 (60.50%)	316 (39.50%)	800 (100.00%)	
Sex	Male	156 (62.15%)	95 (37.85%)	251 (31.37%)	p value: 0.5182 (Pearson's Chi-squared test)
	Female	328 (59.74%)	221 (40.26%)	549 (68.62%)	
	Total	484 (60.50%)	316 (39.50%)	800 (100.00%)	

Age	18-24	59 (56.19%)	46 (43.81%)	105 (13.13%)	
	25-44	296 (61.80%)	183 (38.20%)	479 (59.88%)	p value: 0.5393
	45-65	129 (59.72%)	87 (40.28%)	216 (27.00%)	(Pearson's Chi-squared test)
	Total	484 (60.50%)	316 (39.50%)	800 (100.00%)	
Marital Status[a]	Single	281 (61.49%)	176 (38.51%)	457 (57.13%)	
	Married	178 (61.59%)	111 (38.41%)	289 (36.13%)	p value: 0.1149
	Others	25 (46.30%)	29 (53.70%)	54 (6.75%)	(Pearson's Chi-squared test)
	Total	484 (60.50%)	316 (39.50%)	800 (100.00%)	
Monthly household income[b]	Low	101 (53.44%)	88 (46.56%)	189 (23.68%)	
	Middle	198 (64.08%)	111 (35.92%)	309 (38.72%)	p value: 0.0559
	High	185 (61.67%)	115 (38.33%)	300 (37.59%)	(Pearson's Chi-squared test)
	Total	484 (60.65%)	314 (39.35%)	798 (100.00%)	
Employment status	Full-time	290 (63.46%)	167 (36.54%)	457 (57.13%)	
	Part-time	46 (56.79%)	35 (43.21%)	81 (10.13%)	p value: 0.0863 (Pearson's Chi-squared test)
	Freelance	21 (56.76%)	16 (42.11%)	37 (4.63%)	

Student	62 (63.92%)	35 (36.08%)	97 (12.13%)
Unemployed	65 (50.78%)	63 (49.61%)	128 (16.00%)
Total	484 (60.50%)	316 (39.50%)	800 (100.00%)

a

Others of Marital status include those divorced and widowed.

b

Monthly household income: The low-income group has an income of less than 20,000 Hong Kong dollars (HKD), the middle-income group has an income of HKD 20,000–39,999, and the high-income group has an income of HKD 40,000 or above.

Table S2. Characteristics of participants (N=484).

Name	Category	N(%)
Covariates at individual-level		
Community	Sha Tin	180 (37.19%)
	Central and Western	61 (12.60%)
	Kwun Tong	137 (28.31%)
	Kwai Tsing	106 (21.90%)
Season	Spring	85 (17.56%)
	Summer	31 (6.4%)
	Autumn	123 (25.41%)
	Winter	245 (50.62%)
Sex	Male	156 (32.23%)
	Female	328 (67.77%)
Age	18-24	59 (12.19%)
	25-44	296 (61.16%)
	45-65	129 (26.65%)
Marital Status[a]	Single	281 (58.06%)
	Married	178 (36.78%)
	Others	25 (5.17%)
Education level[b]	Low	78 (16.12%)
	Middle	282 (58.26%)
	High	124 (25.62%)
Monthly household income[c]	Low	101 (20.87%)
	Middle	198 (40.91%)
	High	185 (38.22%)

Employment status	Full-time	290 (59.92%)
	Part-time	46 (9.50%)
	Freelance	21 (4.34%)
	Student	62 (12.81%)
	Unemployed	65 (13.43%)
Household type	Couple with child	246 (50.83%)
	Couple without child	59 (12.19%)
	Single parent	64 (13.22%)
	Others	115 (23.76%)
Overall health status	Poor	68 (14.05%)
	Good	416 (85.95%)
Presence of preexisting sleep problems	Yes	51 (10.54%)
	No	433 (89.46%)
Current smoker	Yes	19 (3.93%)
	No	465 (96.07%)
Current alcohol consumer	Yes	72 (14.88%)
	No	412 (85.12%)
Current coffee consumer	Yes	227 (46.90%)
	No	257 (53.10%)
Current energy-drink consumer	Yes	40 (8.26%)
	No	444 (91.74%)

a

Others of Marital status include those divorced and widowed.

b

Education level: The low group graduated from middle school or lower, the middle group has a bachelor's degree or certification as post-secondary, and the high group has a master's degree or higher.

c

Monthly household income: The low-income group has an income of less than 20,000 Hong Kong dollars (HKD), the middle-income group has an income of HKD 20,000–39,999, and the high-income group has an income of HKD 40,000 or above.

Table S3. Characteristics of night-level variables (N=1748).

Name	Category	N(%)/Mean(SD)
Independent variables		
Astronomical night: IRBM LAN (log lux)		0.73(0.59)
Astronomical night: RMBM LAN (log lux)		0.93(0.56)
Biological night: IRBM LAN (log lux)		0.14(0.38)
Biological night: RMBM LAN (log lux)		0.07(0.24)
Dependent variables		
Sleep duration (h)		6.60 (1.60)
Sleep efficiency (%)		0.73 (0.13)
Sleep onset latency (log h)		0.34 (0.30)
Wake after sleep onset (h)		1.21 (0.85)
Self-reported sleep quality	Poor	236 (13.50%)
	Fair	870 (49.77%)
	Good	642 (36.73%)
Self-reported nighttime awakenings	0	670 (38.33%)
	1	549 (31.41%)
	2	307 (17.56%)
	≥3	222 (12.70%)
Covariates at night-level		
Sleep day type	Weekend	682 (39.02%)
	Weekday	1066 (60.98%)

Wake-up day type	Weekend	506 (28.95%)
	Weekday	1242 (71.05%)
Nap during the daytime	Yes	239 (13.67%)
	No	1509 (86.33%)
Daytime physical activity		1206.2 (412.6)

Table S4. Definition of LAN exposure by IRBM and RMBM in the past literature.

**Definition of light at night**	**Measurement approach (Device;Manufacturer)**	**Data**
Average LAN intensity from 7:00 p.m. to 7:00 a.m. / the entire nighttime (from self-reported bedtime to wake-up time) / 1h post-sleep (60min after self-reported bedtime) / 2h post-sleep / 4h post-sleep / 2h pre-wake (120min before self-reported waketime) / 1h pre-wake	IRBM (portable photometers TES136/TES 1339R; Taishi Electronics Industrial Co., Ltd., Taiwan, China)	a cross-sectional study in Beijing, China[10,11]
1h- and 4h-average intensity of postbedtime light / average light intensity (LANavg) from bedtime to rising time / 1h- and 2h-average intensity of preawake light	IRBM (portable photometers TES1339R/TES 1339R; Taishi Corp., Taiwan, China)	a cross-sectional study in Hefei, China[12]
Average light intensity between self-reported bedtime and rising time / average light intensity between actual sleep period defined by actigraphy / duration of light intensity ≥ 10lux during the self-reported in-bed period / duration of light intensity ≥100lux during the self-reported in-bed period / total time of nighttime light intensity of ≥5 lux	IRBM (light meter LX-28SD; Sato Shouji Inc., Kanagawa,Japan)	The HEIJO-KYO cohort[13–17]
Evening light exposure (average light intensity during the 4h before in-bed time) / Nighttime light exposure (average light intensity during the 2 h after in-bed time)	IRBM (light meter LX-28SD; Sato Shouji Inc., Kanagawa,Japan) for nighttime light exposure; RMBM (wrist-worn Actiwatch 2; Respironics Inc., Murrysville, PA) for evening light exposure	The HEIJO-KYO cohort[18]
The average light intensity between self-reported bedtimes and rising times	IRBM (LX-28SD; Sato Shoji Inc., Kanagawa, Japan)	The APPLE cohort[19,20]

Light exposure within the 5-hour activity nadir	RMBM (wrist-worn GT3X+ ActiGraph; ActiGraph, Pensacola, FL)	NHANES 2011-2014[21]
Average bedroom exposure during the darkest period of the night/ average exposure in the time children were reported to be in bed / average exposure during the time period of civil twilight / average exposure between start of civil twilight and bedtime / average exposure in the hour before children went to bed	RMBM (LightWatcher; Wolf Technologieberatung)	ABCD cohort study[22]
Average short-wavelength LAN power from bedtime to rising time	RMBM (headband-type light sensor G6262; Hamamatsu Photonics K.K., Shizuoka, Japan)	The HEIJO-KYO cohort[23]
Evening light exposure (average light intensity during the 4h before in-bed time)	RMBM (wrist-worn Actillume monitor; Ambulatory Monitoring, Inc., Ardsley, NY)	Participants in the WHI observationa l study[24]
Total light exposure in the 3 hours after sunset	RMBM (wrist-worn ActTrust AT0503; Condor Instruments, São Paulo, SP, Brazil)	a cross-sectional study in Milange and Tengua, Mozambiqu e[25]
Light exposure means during evening hours (between 20:00-05:00)	RMBM (wrist-worn Actiwatch-L; MiniMitter/Respironics, Bend, Oregon, USA)	a cross-sectional study in Minnesota, USA[26]
Night-time light exposure (sunset to 04:00) / Light exposure during standardized night-time (21:00-02:00) / light exposure during the evening (17:00-23:00) and late night (23:00-05:00)	RMBM (HOBO UA-002-08 Pendant Temperature/Light Data Logger)	a cross-sectional study in NYC and NJ, USA[27]

Table S5. Astronomical night: LAN exposure by IRBM and RMBM across geographic contexts.

Name	Community

	Sha Tin (Mean(SD))	Central and Western (Mean(SD))	Kwun Tong (Mean(SD))	Kwai Tsing (Mean(SD))	p value of Kruskal-Wallis rank sum test
IRBM LAN	0.76(0.61)	0.73(0.58)	0.67(0.58)	0.74(0.60)	0.1025
RMBM LAN	0.91(0.57)	0.91(0.58)	0.98(0.57)	0.95(0.52)	0.0824
p value of Wilcoxon rank sum test	<0.001	<0.001	<0.001	<0.001	

Table S6. Astronomical night: LAN exposure by IRBM and RMBM across temporal contexts.

**Name**	**Sleep day type**		**p value of Mann-Whitney U test**	**Wake-up day type**		**p value of Mann-Whitney U test**
	**Weekend (Mean(SD))**	**Weekday (Mean(SD))**		**Weekend (Mean(SD))**	**Weekday (Mean(SD))**	
IRBM LAN	0.75(0.60)	0.71(0.59)	0.1521	0.74(0.60)	0.72(0.59)	0.689
RMBM LAN	0.94(0.56)	0.93(0.56)	0.6997	0.95(0.55)	0.93(0.56)	0.472
p value of Wilcoxon rank sum test	<0.001	<0.001		<0.001	<0.001	

Table S7. Astronomical night: Beta/Odds ratio in LAN exposure to sleep parameters by IRBM and RMBM (95% CI).

	Residence-based			Mobility-based			p-value of Wald tests
	Estimates/ Odds Ratios	CI	p-value	Estimates/ Odds Ratios	CI	p-value	
**Sleep duration**							
Model 1	-0.40	(-0.82,0.03)	0.070	-0.55**	(-0.94,-0.16)	0.006	
Model 2	-0.44*	(-0.86,-0.02)	0.040	-0.55**	(-0.94,-0.16)	0.005	0.702
Model 3	-0.43*	(-0.86,-0.01)	0.044	-0.48**	(-0.87,-0.09)	0.016	0.879
**Sleep efficiency**							
Model 1	-0.03	(-0.07,0.00)	0.054	-0.04*	(-0.07,-0.00)	0.029	
Model 2	-0.03	(-0.07,-0.00)	0.065	-0.03*	(-0.07,-0.00)	0.039	
Model 3	-0.04*	(-0.08,-0.01)	0.012	-0.03*	(-0.07,-0.00)	0.039	0.643
**Sleep onset latency**							
Model 1	0.03	(-0.05,0.11)	0.411	0.07	(-0.00,0.14)	0.055	
Model 2	0.04	(-0.04,0.11)	0.362	0.06	(-0.01,0.13)	0.116	
Model 3	0.05	(-0.03,0.13)	0.197	0.05	(-0.02,0.12)	0.191	
**WASO**							
Model 1	0.00	(-0.22,0.23)	0.972	0.05	(-0.16,0.25)	0.645	
Model 2	-0.01	(-0.24,0.21)	0.924	0.04	(-0.16,0.25)	0.671	
Model 3	0.06	(-0.17,0.28)	0.623	0.07	(-0.14,0.27)	0.506	
**Self-reported nighttime awakenings**							
Model 1	0.39*	(0.18,0.85)	0.019	0.86	(0.43,1.72)	0.669	
Model 2	0.38*	(0.17,0.84)	0.018	0.75	(0.37,1.53)	0.431	
Model 3	0.6	(0.28,1.41)	0.262	1.01	(0.50,2.05)	0.979	
**Self-reported sleep quality**							
Model 1	1.62	(0.72,3.64)	0.241	1.18	(0.57,2.44)	0.653	
Model 2	1.66	(0.74,3.73)	0.222	1.25	(0.60,2.60)	0.55	
Model 3	1.26	(0.57,2.80)	0.573	1.09	(0.53,2.24)	0.820	

Note. Model 1: LAN exposure only; Model 2: Model 1 + nap during the daytime, sleep day type, wake-up day type, daytime physical activity; Model 3: Model 2 + community, season, sex, age, marital status, education level, monthly household income, employment status, household type, overall health status, presence of preexisting sleep problems, current smoker, current alcohol consumer, current coffee consumer, current energy drink consumer. *=p<0.05;**=p<0.01.

Table S8. Astronomical night: stratified analysis for sleep duration by IRBM.

Predictors	Null Model	Model 1	Model 2	Model 3	Model 4	Model 5	Model 6
(Intercept)	6.56***	6.66***	7.05***	7.11***	6.96***	6.74***	7.14***
	(0.05)	(0.08)	(0.17)	(0.49)	(0.50)	(0.52)	(0.49)
IRBM LAN		-0.40	-0.44*	-0.43*	0.14	0.12	-0.06
		(0.22)	(0.21)	(0.21)	(0.40)	(0.08)	(0.03)
Nap during the daytime: No			0.37***	0.37***	0.37***	0.38***	0.37***
			(0.11)	(0.11)	(0.11)	(0.11)	(0.11)
Sleep day type: Weekday			-0.00	-0.00	-0.00	-0.00	-0.00
			(0.07)	(0.07)	(0.07)	(0.07)	(0.07)
Wake-up day type: Weekday			-0.60***	-0.60***	-0.60***	-0.60***	-0.60***
			(0.07)	(0.07)	(0.07)	(0.07)	(0.07)
Daytime physical activity			-0.65*	-0.62*	-0.61*	-0.62*	-0.62*
			(0.28)	(0.29)	(0.29)	(0.29)	(0.29)
Season: Summer				-0.52*	-0.54*	-0.53*	-0.53*
				(0.25)	(0.25)	(0.25)	(0.25)
Season: Autumn				-0.35*	-0.35*	-0.34	-0.34
				(0.18)	(0.17)	(0.17)	(0.18)
Season: Winter				-0.17	-0.18	-0.17	-0.17
				(0.15)	(0.15)	(0.15)	(0.15)
Community: Central and Western				0.21	0.19	0.16	0.20
				(0.17)	(0.17)	(0.17)	(0.17)
Community: Kwun Tong				0.09	0.09	0.08	0.09
				(0.13)	(0.13)	(0.13)	(0.13)
Community: Kwai Tsing				-0.10	-0.10	-0.10	-0.12
				(0.15)	(0.15)	(0.15)	(0.15)
Overall health status. L				0.02	0.03	0.03	0.03
				(0.11)	(0.11)	(0.11)	(0.11)
Presence of preexisting sleep problems: No				0.31	0.32	0.30	0.32
				(0.18)	(0.18)	(0.18)	(0.18)
Sex: Female				0.05	0.25	0.06	0.05
				(0.11)	(0.16)	(0.11)	(0.11)
Age: 25-44				-0.39	-0.38	0.00	-0.39
				(0.21)	(0.21)	(0.28)	(0.21)
Age: 45-65				-0.65**	-0.64**	-0.15	-0.64*
				(0.25)	(0.25)	(0.31)	(0.25)
Marital Status: Others				0.01	0.01	0.03	0.00

	(0.25)	(0.25)	(0.25)	(0.25)
Marital Status: Married	-0.00	-0.01	-0.02	-0.01
	(0.14)	(0.14)	(0.14)	(0.14)
Education level. L	-0.06	-0.07	-0.05	-0.06
	(0.15)	(0.15)	(0.15)	(0.15)
Education level. Q	0.16	0.17	0.17	0.17
	(0.10)	(0.10)	(0.10)	(0.10)
Monthly household income. L	-0.26*	-0.25*	-0.24*	-0.18
	(0.11)	(0.11)	(0.11)	(0.15)
Monthly household income. Q	0.16	0.17	0.15	0.17
	(0.09)	(0.09)	(0.09)	(0.13)
Employment status: Part-time	0.00	0.02	0.03	-0.00
	(0.19)	(0.18)	(0.18)	(0.19)
Employment status: Freelance	-0.21	-0.18	-0.20	-0.21
	(0.26)	(0.25)	(0.25)	(0.25)
Employment status: Student	-0.16	-0.17	-0.19	-0.17
	(0.21)	(0.21)	(0.21)	(0.21)
Employment status: Unemployed	0.06	0.06	0.07	0.06
	(0.18)	(0.18)	(0.18)	(0.18)
Household type: Couple without child	0.12	0.14	0.12	0.11
	(0.17)	(0.17)	(0.17)	(0.17)
Household type: Single parent	-0.08	-0.09	-0.08	-0.09
	(0.17)	(0.17)	(0.17)	(0.17)
Household type: Others	-0.22	-0.22	-0.20	-0.22
	(0.14)	(0.14)	(0.14)	(0.14)
Current smoker: No	0.25	0.23	0.27	0.24
	(0.29)	(0.29)	(0.29)	(0.29)
Current alcohol consumer: No	0.09	0.11	0.08	0.09
	(0.14)	(0.14)	(0.14)	(0.14)
Current coffee consumer: No	0.10	0.09	0.10	0.10
	(0.10)	(0.10)	(0.10)	(0.10)
Current energy drink consumer: No	-0.02	-0.01	-0.02	-0.03
	(0.19)	(0.19)	(0.19)	(0.19)
IRBM LAN: Sex Female		-0.79		

					(0.47)		
IRBM LAN: Age 25-44						-0.20*	
						(0.09)	
IRBM LAN: Age 45-65						-0.28**	
						(0.10)	
IRBM LAN: Income. L							-0.32
							(0.42)
IRBM LAN: Income. Q							-0.03
							(0.36)
AIC	6435.95	6434.66	6362.92	6378.34	6377.53	6374.75	6381.69
BIC	6452.34	6456.52	6406.65	6575.12	6579.78	6582.47	6589.41
Log Likelihood	-3214.97	-3213.33	-3173.46	-3153.17	-3151.77	-3149.37	-3152.85
ICC	0.29	0.29	0.29	0.26	0.25	0.25	0.26
Marginal R^2/Conditional R^2	0.000/0.286	0.003/0.289	0.040/0.317	0.081/0.317	0.084/0.315	0.088/0.320	0.082/0.316
Var: ID (Intercept)	0.74	0.74	0.71	0.60	0.59	0.59	0.60
Var: Residual	1.83	1.83	1.75	1.75	1.75	1.74	1.75

$^{***}p < 0.001$; $^{**}p < 0.01$; $^{*}p < 0.05$

Table S9. Astronomical night: stratified analysis for sleep duration by RMBM.

Predictors	Null Model	Model 1	Model 2	Model 3	Model 4
(Intercept)	6.56***	6.76***	7.08***	7.14***	7.21***
	(0.05)	(0.09)	(0.17)	(0.49)	(0.50)
RMBM LAN		-0.55**	-0.55**	-0.48**	-0.74*
		(0.20)	(0.20)	(0.20)	(0.34)
Nap during the daytime: No			0.37***	0.37***	0.37***
			(0.11)	(0.11)	(0.11)
Sleep day type: Weekday			-0.00	0.00	0.00
			(0.07)	(0.07)	(0.07)
Wake-up day type: Weekday			-0.61***	-0.60***	-0.60***
			(0.07)	(0.07)	(0.07)
Daytime physical activity			-0.52	-0.50	-0.48
			(0.28)	(0.29)	(0.29)
Season: Summer				-0.48	-0.47
				(0.25)	(0.25)
Season: Autumn				-0.36*	-0.35*
				(0.17)	(0.17)
Season: Winter				-0.19	-0.18
				(0.15)	(0.15)
Community: Central and Western				0.22	0.22
				(0.17)	(0.17)
Community: Kwun Tong				0.12	0.12
				(0.13)	(0.13)
Community: Kwai Tsing				-0.08	-0.08
				(0.15)	(0.15)
Overall health status. L				0.01	0.02
				(0.11)	(0.11)
Presence of preexisting sleep problems: No				0.33	0.32
				(0.18)	(0.18)
Sex: Female				0.03	-0.13
				(0.11)	(0.19)
Age: 25-44				-0.37	-0.36
				(0.21)	(0.21)
Age: 45-65				-0.64**	-0.63**
				(0.25)	(0.25)
Marital Status: Others				0.04	0.03
				(0.25)	(0.25)
Marital Status: Married				0.03	0.03
				(0.14)	(0.14)
Education level. L				-0.05	-0.05
				(0.15)	(0.15)
Education level. Q				0.16	0.16

				(0.10)	(0.10)
Monthly household income. L				-0.26*	-0.26*
				(0.11)	(0.11)
Monthly household income. Q				0.16	0.15
				(0.09)	(0.09)
Employment status: Part-time				0.00	0.00
				(0.19)	(0.19)
Employment status: Freelance				-0.22	-0.23
				(0.25)	(0.25)
Employment status: Student				-0.16	-0.15
				(0.21)	(0.21)
Employment status: Unemployed				0.04	0.04
				(0.18)	(0.18)
Household type: Couple without child				0.12	0.12
				(0.17)	(0.17)
Household type: Single parent				-0.08	-0.07
				(0.17)	(0.17)
Household type: Others				-0.21	-0.21
				(0.14)	(0.14)
Current smoker: No				0.25	0.25
				(0.29)	(0.29)
Current alcohol consumer: No				0.09	0.09
				(0.14)	(0.14)
Current coffee consumer: No				0.08	0.09
				(0.10)	(0.10)
Current energy drink consumer: No				-0.04	-0.04
				(0.19)	(0.19)
RMBM LAN: Sex Female					0.40
					(0.41)
AIC	6435.95	6430.47	6359.37	6376.63	6377.70
BIC	6452.34	6452.33	6403.10	6573.41	6579.96
Log Likelihood	-3214.97	-3211.23	-3171.69	-3152.31	-3151.85
ICC	0.29	0.28	0.29	0.26	0.26
Marginal R^2/Conditional R^2	0.000/0.286	0.005/0.286	0.043/0.316	0.083/0.318	0.084/0.318
Var: ID (Intercept)	0.74	0.72	0.70	0.60	0.60
Var: Residual	1.83	1.83	1.75	1.75	1.75

$^{***}p < 0.001$; $^{**}p < 0.01$; $^{*}p < 0.05$

Table S10. Astronomical night: stratified analysis for sleep efficiency by IRBM.

Predictors	Null Model	Model 1	Model 2	Model 3	Model 4	Model 5	Model 6
(Intercept)	0.73***	0.74***	0.73***	0.60***	0.60***	0.61***	0.60***
	(0.00)	(0.01)	(0.01)	(0.04)	(0.04)	(0.04)	(0.04)
IRBM LAN		-0.03	-0.03	-0.04*	-0.04	-0.08	-0.06**
		(0.02)	(0.02)	(0.02)	(0.03)	(0.04)	(0.02)
Nap during the daytime: No			0.00	0.01	0.01	0.01	0.01
			(0.01)	(0.01)	(0.01)	(0.01)	(0.01)
Sleep day type: Weekday			0.01	0.01	0.01	0.01	0.01
			(0.01)	(0.01)	(0.01)	(0.01)	(0.01)
Wake-up day type: Weekday			0.01	0.01*	0.01*	0.01*	0.01*
			(0.01)	(0.01)	(0.01)	(0.01)	(0.01)
Daytime physical activity			-0.01	-0.01	-0.01	-0.01	-0.01
			(0.02)	(0.02)	(0.02)	(0.02)	(0.02)
Season: Summer				0.01	0.01	0.01	0.01
				(0.02)	(0.02)	(0.02)	(0.02)
Season: Autumn				0.03	0.03	0.03	0.03
				(0.02)	(0.02)	(0.01)	(0.02)
Season: Winter				0.04**	0.04**	0.04**	0.04**
				(0.01)	(0.01)	(0.01)	(0.01)
Community: Central and Western				-0.01	-0.01	-0.00	-0.00
				(0.01)	(0.01)	(0.01)	(0.01)
Community: Kwun Tong				-0.02	-0.02	-0.02	-0.02
				(0.01)	(0.01)	(0.01)	(0.01)
Community: Kwai Tsing				-0.00	-0.00	-0.00	-0.00
				(0.01)	(0.01)	(0.01)	(0.01)
Overall health status. L				0.01	0.01	0.01	0.01
				(0.01)	(0.01)	(0.01)	(0.01)
Presence of preexisting sleep problems: No				0.01	0.01	0.01	0.01
				(0.02)	(0.02)	(0.02)	(0.02)
Sex: Female				0.03***	0.04*	0.03***	0.03***
				(0.01)	(0.01)	(0.01)	(0.01)
Age: 25-44				-0.01	-0.01	-0.03	-0.01
				(0.02)	(0.02)	(0.02)	(0.02)
Age: 45-65				-0.02	-0.02	-0.02	-0.02
				(0.02)	(0.02)	(0.03)	(0.02)
Marital Status: Others				-0.01	-0.01	-0.00	-0.01
				(0.02)	(0.02)	(0.02)	(0.02)
Marital Status: Married				0.01	0.01	0.01	0.01
				(0.01)	(0.01)	(0.01)	(0.01)

Education level. L	0.01	0.01	0.01	0.01
	(0.01)	(0.01)	(0.01)	(0.01)
Education level. Q	0.00	0.00	0.00	0.00
	(0.01)	(0.01)	(0.01)	(0.01)
Monthly household income. L	0.02	0.02	0.02	0.00
	(0.01)	(0.01)	(0.01)	(0.01)
Monthly household income. Q	0.00	0.00	0.00	0.01
	(0.01)	(0.01)	(0.01)	(0.01)
Employment status: Part-time	0.02	0.03	0.02	0.03
	(0.02)	(0.02)	(0.02)	(0.02)
Employment status: Freelance	-0.02	-0.02	-0.02	-0.02
	(0.02)	(0.02)	(0.02)	(0.02)
Employment status: Student	-0.01	-0.01	-0.01	-0.01
	(0.02)	(0.02)	(0.02)	(0.02)
Employment status: Unemployed	-0.01	-0.01	-0.01	-0.01
	(0.02)	(0.02)	(0.02)	(0.02)
Household type: Couple without child	-0.00	-0.00	-0.00	0.00
	(0.01)	(0.01)	(0.01)	(0.01)
Household type: Single parent	0.03	0.03	0.03	0.03*
	(0.01)	(0.01)	(0.01)	(0.01)
Household type: Others	0.02	0.02	0.02	0.02
	(0.01)	(0.01)	(0.01)	(0.01)
Current smoker: No	0.05	0.05	0.05	0.05*
	(0.02)	(0.02)	(0.02)	(0.02)
Current alcohol consumer: No	-0.01	-0.01	-0.01	-0.01
	(0.01)	(0.01)	(0.01)	(0.01)
Current coffee consumer: No	0.01	0.01	0.01	0.01
	(0.01)	(0.01)	(0.01)	(0.01)
Current energy drink consumer: No	0.03	0.03	0.03	0.03
	(0.02)	(0.02)	(0.02)	(0.02)
IRBM LAN: Sex Female		-0.01		
		(0.04)		
IRBM LAN: Age 25-44			0.06	
			(0.05)	
IRBM LAN: Age 45-65			-0.01	

						(0.06)	
IRBM LAN: Income. L							0.06
							(0.03)
IRBM LAN: Income. Q							-0.04
							(0.03)
AIC	-2473.25	-2474.95	-2475.35	-2474.95	-2473.00	-2474.55	-2474.26
BIC	-2456.85	-2453.09	-2431.62	-2278.17	-2270.75	-2266.83	-2266.54
Log Likelihood	1239.62	1241.47	1245.68	1273.48	1273.50	1275.27	1275.13
ICC	0.37	0.38	0.38	0.33	0.33	0.33	0.33
Marginal R^2/Conditional R^2	0.000/0.374	0.003/0.377	0.006/0.381	0.067/0.379	0.067/0.379	0.070/0.377	0.069/0.379
Var: ID (Intercept)	0.01	0.01	0.01	0.01	0.01	0.01	0.01
Var: Residual	0.01	0.01	0.01	0.01	0.01	0.01	0.01

$^{***}p < 0.001$; $^{**}p < 0.01$; $^{*}p < 0.05$

Table S11. Astronomical night: stratified analysis for sleep efficiency by RMBM.

Predictors	Null Model	Model 1	Model 2	Model 3	Model 4
(Intercept)	0.73***<br>(0.00)	0.75***<br>(0.01)	0.73***<br>(0.01)	0.60***<br>(0.04)	0.60***<br>(0.04)
RMBM LAN		-0.04*<br>(0.02)	-0.03*<br>(0.02)	-0.03*<br>(0.02)	-0.05<br>(0.03)
Nap during the daytime: No			0.00<br>(0.01)	0.01<br>(0.01)	0.01<br>(0.01)
Sleep day type: Weekday			0.01<br>(0.01)	0.01<br>(0.01)	0.01<br>(0.01)
Wake-up day type: Weekday			0.01<br>(0.01)	0.01*<br>(0.01)	0.01*<br>(0.01)
Daytime physical activity			-0.00<br>(0.02)	-0.00<br>(0.02)	-0.00<br>(0.02)
Season: Summer				0.02<br>(0.02)	0.02<br>(0.02)
Season: Autumn				0.03<br>(0.01)	0.03<br>(0.01)
Season: Winter				0.04**<br>(0.01)	0.04**<br>(0.01)
Community: Central and Western				-0.01<br>(0.01)	-0.01<br>(0.01)
Community: Kwun Tong				-0.02<br>(0.01)	-0.02<br>(0.01)
Community: Kwai Tsing				-0.00<br>(0.01)	-0.00<br>(0.01)
Overall health status. L				0.01<br>(0.01)	0.01<br>(0.01)
Presence of preexisting sleep problems: No				0.01<br>(0.02)	0.01<br>(0.02)
Sex: Female				0.03**<br>(0.01)	0.02<br>(0.02)
Age: 25-44				-0.01<br>(0.02)	-0.01<br>(0.02)
Age: 45-65				-0.02<br>(0.02)	-0.02<br>(0.02)
Marital Status: Others				-0.00<br>(0.02)	-0.00<br>(0.02)
Marital Status: Married				0.01<br>(0.01)	0.01<br>(0.01)
Education level. L				0.01<br>(0.01)	0.01<br>(0.01)
Education level. Q				0.00	0.00

				(0.01)	(0.01)
Monthly household income. L				0.01	0.01
				(0.01)	(0.01)
Monthly household income. Q				0.00	0.00
				(0.01)	(0.01)
Employment status: Part-time				0.02	0.02
				(0.02)	(0.02)
Employment status: Freelance				-0.02	-0.02
				(0.02)	(0.02)
Employment status: Student				-0.01	-0.01
				(0.02)	(0.02)
Employment status: Unemployed				-0.01	-0.01
				(0.02)	(0.02)
Household type: Couple without child				-0.00	-0.00
				(0.01)	(0.01)
Household type: Single parent				0.03	0.03
				(0.01)	(0.01)
Household type: Others				0.02	0.02
				(0.01)	(0.01)
Current smoker: No				0.05	0.05
				(0.02)	(0.02)
Current alcohol consumer: No				-0.01	-0.01
				(0.01)	(0.01)
Current coffee consumer: No				0.01	0.01
				(0.01)	(0.01)
Current energy drink consumer: No				0.03	0.03
				(0.02)	(0.02)
RMBM LAN: Sex Female					0.02
					(0.03)
AIC	-2473.25	-2476.00	-2476.20	-2472.85	-2471.28
BIC	-2456.85	-2454.14	-2432.47	-2276.07	-2269.03
Log Likelihood	1239.62	1242.00	1246.10	1272.42	1272.64
ICC	0.37	0.37	0.37	0.33	0.33
Marginal R^2/Conditional R^2	0.000/0.374	0.003/0.372	0.006/0.376	0.066/0.375	0.066/0.375
Var: ID (Intercept)	0.01	0.01	0.01	0.01	0.01
Var: Residual	0.01	0.01	0.01	0.01	0.01

$^{***}p < 0.001$; $^{**}p < 0.01$; $^{*}p < 0.05$

Table S12. Astronomical night: stratified analysis for sleep onset latency by IRBM.

Predictors	Null Model	Model 1	Model 2	Model 3	Model 4
(Intercept)	0.34***	0.33***	0.35***	0.49***	0.48***
	(0.01)	(0.01)	(0.03)	(0.09)	(0.09)
IRBM LAN		0.03	0.04	0.05	0.10
		(0.04)	(0.04)	(0.04)	(0.07)
Nap during the daytime: No			-0.02	-0.02	-0.02
			(0.02)	(0.02)	(0.02)
Sleep day type: Weekday			-0.02	-0.01	-0.01
			(0.01)	(0.01)	(0.01)
Wake-up day type: Weekday			-0.06***	-0.06***	-0.06***
			(0.01)	(0.01)	(0.01)
Daytime physical activity			0.12*	0.12*	0.12*
			(0.05)	(0.06)	(0.06)
Season: Summer				-0.03	-0.03
				(0.04)	(0.04)
Season: Autumn				-0.02	-0.02
				(0.03)	(0.03)
Season: Winter				-0.05*	-0.05*
				(0.03)	(0.03)
Community: Central and Western				0.01	0.01
				(0.03)	(0.03)
Community: Kwun Tong				0.02	0.02
				(0.02)	(0.02)
Community: Kwai Tsing				0.01	0.01
				(0.03)	(0.03)
Overall health status. L				-0.03	-0.03
				(0.02)	(0.02)
Presence of preexisting sleep problems: No				0.03	0.03
				(0.03)	(0.03)
Sex: Female				-0.02	-0.00
				(0.02)	(0.03)
Age: 25-44				0.01	0.01
				(0.04)	(0.04)
Age: 45-65				-0.00	-0.00
				(0.05)	(0.05)
Marital Status: Others				-0.02	-0.02
				(0.04)	(0.04)
Marital Status: Married				-0.01	-0.01
				(0.03)	(0.03)
Education level. L				-0.04	-0.04
				(0.03)	(0.03)
Education level. Q				-0.01	-0.01

				(0.02)	(0.02)
Monthly household income. L				0.00	0.01
				(0.02)	(0.02)
Monthly household income. Q				0.00	0.00
				(0.02)	(0.02)
Employment status: Part-time				-0.02	-0.02
				(0.03)	(0.03)
Employment status: Freelance				0.07	0.07
				(0.05)	(0.05)
Employment status: Student				0.02	0.02
				(0.04)	(0.04)
Employment status: Unemployed				-0.01	-0.01
				(0.03)	(0.03)
Household type: Couple without child				-0.01	-0.01
				(0.03)	(0.03)
Household type: Single parent				-0.04	-0.04
				(0.03)	(0.03)
Household type: Others				-0.02	-0.02
				(0.02)	(0.02)
Current smoker: No				-0.00	-0.01
				(0.05)	(0.05)
Current alcohol consumer: No				0.01	0.01
				(0.03)	(0.03)
Current coffee consumer: No				-0.05*	-0.05**
				(0.02)	(0.02)
Current energy drink consumer: No				-0.09**	-0.09**
				(0.03)	(0.03)
IRBM LAN: Sex Female					-0.06
					(0.09)
AIC	631.20	632.53	617.47	638.59	640.11
BIC	647.60	654.40	661.20	835.38	842.36
Log Likelihood	-312.60	-312.27	-300.74	-283.30	-283.05
ICC	0.20	0.20	0.21	0.19	0.19
Marginal R^2/Conditional R^2	0.000/0.203	0.001/0.204	0.013/0.219	0.043/0.221	0.043/0.221
Var: ID (Intercept)	0.02	0.02	0.02	0.02	0.02
Var: Residual	0.07	0.07	0.07	0.07	0.07

$^{***}p < 0.001$; $^{**}p < 0.01$; $^{*}p < 0.05$

Table S13. Astronomical night: stratified analysis for sleep onset latency by RMBM.

Predictors	Null Model	Model 1	Model 2	Model 3	Model 4
(Intercept)	0.34***	0.32***	0.34***	0.49***	0.48***
	(0.01)	(0.02)	(0.03)	(0.09)	(0.09)
RMBM LAN		0.07	0.06	0.05	0.10
		(0.04)	(0.04)	(0.04)	(0.06)
Nap during the daytime: No			-0.02	-0.02	-0.02
			(0.02)	(0.02)	(0.02)
Sleep day type: Weekday			-0.02	-0.01	-0.01
			(0.01)	(0.01)	(0.01)
Wake-up day type: Weekday			-0.06***	-0.06***	-0.06***
			(0.01)	(0.01)	(0.01)
Daytime physical activity			0.10	0.10	0.10
			(0.05)	(0.06)	(0.06)
Season: Summer				-0.04	-0.04
				(0.05)	(0.04)
Season: Autumn				-0.02	-0.02
				(0.03)	(0.03)
Season: Winter				-0.05	-0.05*
				(0.03)	(0.03)
Community: Central and Western				0.01	0.01
				(0.03)	(0.03)
Community: Kwun Tong				0.02	0.02
				(0.02)	(0.02)
Community: Kwai Tsing				0.01	0.00
				(0.03)	(0.03)
Overall health status. L				-0.03	-0.03
				(0.02)	(0.02)
Presence of preexisting sleep problems: No				0.03	0.03
				(0.03)	(0.03)
Sex: Female				-0.02	0.01
				(0.02)	(0.04)
Age: 25-44				0.01	0.01
				(0.04)	(0.04)
Age: 45-65				-0.00	-0.00
				(0.05)	(0.05)
Marital Status: Others				-0.03	-0.03
				(0.04)	(0.04)
Marital Status: Married				-0.01	-0.01
				(0.03)	(0.03)
Education level. L				-0.04	-0.04
				(0.03)	(0.03)
Education level. Q				-0.01	-0.01

				(0.02)	(0.02)
Monthly household income. L				0.01	0.01
				(0.02)	(0.02)
Monthly household income. Q				0.00	0.00
				(0.02)	(0.02)
Employment status: Part-time				-0.02	-0.02
				(0.03)	(0.03)
Employment status: Freelance				0.07	0.07
				(0.05)	(0.05)
Employment status: Student				0.02	0.02
				(0.04)	(0.04)
Employment status: Unemployed				-0.01	-0.01
				(0.03)	(0.03)
Household type: Couple without child				-0.01	-0.01
				(0.03)	(0.03)
Household type: Single parent				-0.04	-0.04
				(0.03)	(0.03)
Household type: Others				-0.02	-0.02
				(0.02)	(0.02)
Current smoker: No				-0.00	-0.01
				(0.05)	(0.05)
Current alcohol consumer: No				0.01	0.01
				(0.03)	(0.03)
Current coffee consumer: No				-0.05^{*}	-0.05^{*}
				(0.02)	(0.02)
Current energy drink consumer: No				-0.09^{**}	-0.09^{**}
				(0.03)	(0.03)
RMBM LAN: Sex Female					-0.07
					(0.08)
AIC	631.20	629.53	615.84	638.55	639.66
BIC	647.60	651.39	659.57	835.33	841.91
Log Likelihood	-312.60	-310.76	-299.92	-283.27	-282.83
ICC	0.20	0.20	0.21	0.19	0.19
Marginal R^2/Conditional R^2	0.000/0.203	0.003/0.205	0.014/0.219	0.043/0.219	0.043/0.220
Var: ID (Intercept)	0.02	0.02	0.02	0.02	0.02
Var: Residual	0.07	0.07	0.07	0.07	0.07

$^{***}p < 0.001$; $^{**}p < 0.01$; $^{*}p < 0.05$

Table S14. Astronomical night: stratified analysis for WASO by IRBM.

Predictors	Null Model	Model 1	Model 2	Model 3	Model 4
(Intercept)	1.19*** (0.03)	1.19*** (0.04)	1.29*** (0.09)	2.13*** (0.26)	2.07*** (0.27)
IRBM LAN		0.00 (0.02)	-0.01 (0.11)	0.06 (0.11)	0.27 (0.21)
Nap during the daytime: No			0.14* (0.06)	0.12* (0.06)	0.12* (0.06)
Sleep day type: Weekday			-0.02 (0.03)	-0.03 (0.03)	-0.03 (0.03)
Wake-up day type: Weekday			-0.19*** (0.04)	-0.19*** (0.04)	-0.19*** (0.04)
Daytime physical activity			-0.17 (0.15)	-0.13 (0.15)	-0.12 (0.15)
Season: Summer				-0.18 (0.14)	-0.19 (0.14)
Season: Autumn				-0.25** (0.09)	-0.25** (0.09)
Season: Winter				-0.23** (0.08)	-0.23** (0.08)
Community: Central and Western				0.12 (0.09)	0.11 (0.09)
Community: Kwun Tong				0.15* (0.07)	0.15* (0.07)
Community: Kwai Tsing				-0.06 (0.08)	-0.06 (0.08)
Overall health status. L				-0.06 (0.06)	-0.06 (0.06)
Presence of preexisting sleep problems: No				-0.02 (0.10)	-0.02 (0.10)
Sex: Female				-0.24*** (0.06)	-0.16 (0.09)
Age: 25-44				-0.15 (0.12)	-0.15 (0.12)
Age: 45-65				-0.16 (0.14)	-0.16 (0.14)
Marital Status: Others				0.09 (0.14)	0.09 (0.14)
Marital Status: Married				-0.10 (0.08)	-0.11 (0.08)
Education level. L				-0.05 (0.08)	-0.05 (0.08)
Education level. Q				0.05	0.05

				(0.05)	(0.05)
Monthly household income. L				-0.18**	-0.18**
				(0.06)	(0.06)
Monthly household income. Q				0.07	0.07
				(0.05)	(0.05)
Employment status: Part-time				-0.09	-0.09
				(0.10)	(0.10)
Employment status: Freelance				-0.02	-0.01
				(0.14)	(0.14)
Employment status: Student				-0.07	-0.07
				(0.11)	(0.11)
Employment status: Unemployed				0.11	0.11
				(0.10)	(0.10)
Household type: Couple without child				0.09	0.10
				(0.09)	(0.09)
Household type: Single parent				-0.17	-0.17
				(0.09)	(0.09)
Household type: Others				-0.13	-0.13
				(0.07)	(0.07)
Current smoker: No				-0.28	-0.29
				(0.16)	(0.15)
Current alcohol consumer: No				0.10	0.10
				(0.08)	(0.08)
Current coffee consumer: No				0.03	0.02
				(0.06)	(0.06)
Current energy drink consumer: No				-0.06	-0.06
				(0.10)	(0.10)
IRBM LAN: Sex Female					-0.30
					(0.25)
AIC	4103.34	4105.34	4080.49	4072.41	4072.96
BIC	4119.74	4127.20	4124.22	4269.19	4275.21
Log Likelihood	-2048.67	-2048.67	-2032.25	-2000.20	-1999.48
ICC	0.35	0.35	0.35	0.30	0.30
Marginal R^2/Conditional R^2	0.000/0.346	0.000/0.346	0.014/0.362	0.086/0.364	0.087/0.363
Var: ID (Intercept)	0.24	0.24	0.25	0.20	0.20
Var: Residual	0.46	0.46	0.45	0.45	0.45

$^{***}p < 0.001$; $^{**}p < 0.01$; $^{*}p < 0.05$

Table S15. Astronomical night: stratified analysis for WASO by RMBM.

Predictors	Null Model	Model 1	Model 2	Model 3	Model 4
(Intercept)	1.19***	1.18***	1.27***	2.13***	2.14***
	(0.03)	(0.05)	(0.09)	(0.26)	(0.27)
RMBM LAN		0.05	0.04	0.07	0.02
		(0.10)	(0.10)	(0.10)	(0.18)
Nap during the daytime: No			0.14*	0.12*	0.12*
			(0.06)	(0.06)	(0.06)
Sleep day type: Weekday			-0.02	-0.03	-0.03
			(0.03)	(0.03)	(0.03)
Wake-up day type: Weekday			-0.19***	-0.19***	-0.19***
			(0.04)	(0.04)	(0.04)
Daytime physical activity			-0.18	-0.15	-0.14
			(0.15)	(0.15)	(0.15)
Season: Summer				-0.19	-0.18
				(0.14)	(0.14)
Season: Autumn				-0.25**	-0.25**
				(0.09)	(0.09)
Season: Winter				-0.23**	-0.23**
				(0.08)	(0.08)
Community: Central and Western				0.12	0.12
				(0.09)	(0.09)
Community: Kwun Tong				0.15*	0.15*
				(0.07)	(0.07)
Community: Kwai Tsing				-0.06	-0.06
				(0.08)	(0.08)
Overall health status. L				-0.06	-0.05
				(0.06)	(0.06)
Presence of preexisting sleep problems: No				-0.02	-0.02
				(0.10)	(0.10)
Sex: Female				-0.23***	-0.26**
				(0.06)	(0.10)
Age: 25-44				-0.15	-0.15
				(0.12)	(0.12)
Age: 45-65				-0.16	-0.16
				(0.14)	(0.14)
Marital Status: Others				0.09	0.09
				(0.14)	(0.14)
Marital Status: Married				-0.11	-0.11
				(0.08)	(0.08)
Education level. L				-0.05	-0.05
				(0.08)	(0.08)
Education level. Q				0.05	0.06

				(0.05)	(0.05)
Monthly household income. L				-0.18**	-0.18**
				(0.06)	(0.06)
Monthly household income. Q				0.07	0.07
				(0.05)	(0.05)
Employment status: Part-time				-0.09	-0.09
				(0.10)	(0.10)
Employment status: Freelance				-0.01	-0.02
				(0.14)	(0.14)
Employment status: Student				-0.07	-0.07
				(0.11)	(0.11)
Employment status: Unemployed				0.11	0.11
				(0.10)	(0.10)
Household type: Couple without child				0.09	0.09
				(0.09)	(0.09)
Household type: Single parent				-0.17	-0.17
				(0.09)	(0.09)
Household type: Others				-0.14	-0.13
				(0.07)	(0.07)
Current smoker: No				-0.29	-0.28
				(0.16)	(0.15)
Current alcohol consumer: No				0.10	0.10
				(0.08)	(0.08)
Current coffee consumer: No				0.03	0.03
				(0.06)	(0.06)
Current energy drink consumer: No				-0.06	-0.06
				(0.10)	(0.10)
RMBM LAN: Sex Female					0.08
					(0.22)
AIC	4103.34	4105.13	4080.32	4072.21	4074.08
BIC	4119.74	4126.99	4124.05	4268.99	4276.33
Log Likelihood	-2048.67	-2048.56	-2032.16	-2000.10	-2000.04
ICC	0.35	0.35	0.35	0.30	0.31
Marginal R^2/Conditional R^2	0.000/0.346	0.000/0.346	0.014/0.361	0.086/0.363	0.086/0.363
Var: ID (Intercept)	0.24	0.24	0.24	0.20	0.20
Var: Residual	0.46	0.46	0.45	0.45	0.45

$^{***}p < 0.001$; $^{**}p < 0.01$; $^{*}p < 0.05$

Table S16. Astronomical night: stratified analysis for self-reported nighttime awakenings by IRBM.

Predictors	Null Model	Model 1	Model 2	Model 3	Model 4
0 | 1	-0.88***	-1.12***	-1.75***	-2.26*	-2.54*
	(0.12)	(0.16)	(0.32)	(1.06)	(1.07)
1 | 2	1.32***	1.08***	0.53	0.03	-0.26
	(0.13)	(0.16)	(0.32)	(1.06)	(1.07)
2 | ≥3	3.07***	2.83***	2.35***	1.84	1.56
	(0.15)	(0.18)	(0.32)	(1.06)	(1.07)
IRBM LAN		-0.95*	-0.98*	-0.46	-1.51
		(0.40)	(0.41)	(0.41)	(0.78)
Nap during the daytime: No			0.01	-0.01	-0.01
			(0.19)	(0.19)	(0.19)
Sleep day type: Weekday			-0.15	-0.14	-0.15
			(0.11)	(0.11)	(0.11)
Wake-up day type: Weekday			-0.82***	-0.82***	-0.83***
			(0.12)	(0.12)	(0.12)
Daytime physical activity			0.30	-0.11	-0.13
			(0.51)	(0.51)	(0.51)
Season: Summer				-1.27*	-1.24*
				(0.56)	(0.56)
Season: Autumn				-0.55	-0.53
				(0.39)	(0.39)
Season: Winter				-0.65*	-0.65
				(0.33)	(0.33)
Community: Central and Western				-0.46	-0.43
				(0.39)	(0.39)
Community: Kwun Tong				0.46	0.48
				(0.29)	(0.29)
Community: Kwai Tsing				-0.01	-0.02
				(0.33)	(0.33)
Overall health status. L				-0.82***	-0.83***
				(0.24)	(0.24)
Presence of preexisting sleep problems: No				0.24	0.23
				(0.39)	(0.39)
Sex: Female				0.32	-0.04
				(0.25)	(0.34)
Age: 25-44				0.72	0.71
				(0.48)	(0.48)
Age: 45-65				1.20*	1.19*
				(0.57)	(0.57)
Marital Status: Others				0.16	0.15
				(0.57)	(0.57)
Marital Status: Married				0.58	0.61

				(0.32)	(0.32)
Education level. L				0.40	0.41
				(0.33)	(0.33)
Education level. Q				0.08	0.08
				(0.21)	(0.21)
Monthly household income. L				-0.30	-0.31
				(0.25)	(0.25)
Monthly household income. Q				0.10	0.09
				(0.20)	(0.20)
Employment status: Part-time				-0.02	-0.04
				(0.41)	(0.41)
Employment status: Freelance				-0.83	-0.90
				(0.57)	(0.57)
Employment status: Student				-0.32	-0.30
				(0.47)	(0.47)
Employment status: Unemployed				0.67	0.67
				(0.41)	(0.41)
Household type: Couple without child				0.82*	0.78*
				(0.38)	(0.38)
Household type: Single parent				0.14	0.17
				(0.38)	(0.38)
Household type: Others				0.28	0.28
				(0.31)	(0.31)
Current smoker: No				-0.54	-0.52
				(0.63)	(0.63)
Current alcohol consumer: No				-0.61	-0.65*
				(0.33)	(0.33)
Current coffee consumer: No				0.14	0.16
				(0.23)	(0.23)
Current energy drink consumer: No				-0.18	-0.20
				(0.42)	(0.42)
IRBM LAN: Sex Female					1.44
					(0.91)
AIC	4020.23	4016.68	3971.21	3954.82	3954.28
BIC	4042.09	4044.01	4020.41	4157.07	4161.99
Log Likelihood	-2006.11	-2003.34	-1976.60	-1940.41	-1939.14
ICC	0.59	0.59	0.61	0.56	0.56
Marginal R^2/Conditional R^2	0.000/0.588	0.005/0.587	0.023/0.615	0.137/0.621	0.140/0.622
Var: ID (Intercept)	4.70	4.65	5.06	4.26	4.25

$^{***}p < 0.001$; $^{**}p < 0.01$; $^{*}p < 0.05$

Table S17. Astronomical night: stratified analysis for self-reported nighttime awakenings by RMBM.

Predictors	Null Model	Model 1	Model 2	Model 3	Model 4
0 | 1	-0.88***	-0.93***	-1.53***	-2.13*	-2.26*
	(0.12)	(0.18)	(0.31)	(1.05)	(1.07)
1 | 2	1.32***	1.27***	0.75*	0.15	0.02
	(0.13)	(0.18)	(0.31)	(1.05)	(1.06)
2 | ≥3	3.07***	3.02***	2.56***	1.97	1.84
	(0.15)	(0.20)	(0.32)	(1.05)	(1.07)
RMBM LAN		-0.15	-0.29	0.01	-0.46
		(0.35)	(0.36)	(0.36)	(0.64)
Nap during the daytime: No			0.02	-0.01	-0.01
			(0.19)	(0.19)	(0.19)
Sleep day type: Weekday			-0.14	-0.14	-0.14
			(0.11)	(0.11)	(0.11)
Wake-up day type: Weekday			-0.82***	-0.82***	-0.83***
			(0.12)	(0.12)	(0.12)
Daytime physical activity			0.45	-0.08	-0.05
			(0.51)	(0.52)	(0.52)
Season: Summer				-1.30*	-1.29*
				(0.57)	(0.57)
Season: Autumn				-0.56	-0.55
				(0.39)	(0.39)
Season: Winter				-0.67*	-0.66*
				(0.33)	(0.33)
Community: Central and Western				-0.46	-0.46
				(0.39)	(0.39)
Community: Kwun Tong				0.49	0.49
				(0.29)	(0.29)
Community: Kwai Tsing				0.00	0.01
				(0.33)	(0.33)
Overall health status. L				-0.83***	-0.82***
				(0.24)	(0.24)
Presence of preexisting sleep problems: No				0.26	0.25
				(0.39)	(0.39)
Sex: Female				0.32	0.06
				(0.25)	(0.38)
Age: 25-44				0.72	0.74
				(0.48)	(0.48)
Age: 45-65				1.22*	1.23*
				(0.57)	(0.57)
Marital Status: Others				0.15	0.15
				(0.57)	(0.57)
Marital Status: Married				0.61	0.61

				(0.32)	(0.32)
Education level. L				0.40	0.40
				(0.33)	(0.33)
Education level. Q				0.07	0.08
				(0.21)	(0.22)
Monthly household income. L				-0.32	-0.33
				(0.25)	(0.25)
Monthly household income. Q				0.11	0.11
				(0.20)	(0.20)
Employment status: Part-time				-0.02	-0.02
				(0.41)	(0.42)
Employment status: Freelance				-0.86	-0.87
				(0.57)	(0.57)
Employment status: Student				-0.34	-0.33
				(0.47)	(0.47)
Employment status: Unemployed				0.67	0.67
				(0.41)	(0.41)
Household type: Couple without child				0.84*	0.85*
				(0.38)	(0.38)
Household type: Single parent				0.14	0.15
				(0.38)	(0.38)
Household type: Others				0.29	0.29
				(0.31)	(0.31)
Current smoker: No				-0.57	-0.56
				(0.63)	(0.63)
Current alcohol consumer: No				-0.61	-0.60
				(0.33)	(0.33)
Current coffee consumer: No				0.12	0.13
				(0.23)	(0.23)
Current energy drink consumer: No				-0.19	-0.19
				(0.42)	(0.42)
RMBM LAN: Sex Female					0.68
					(0.77)
AIC	4020.23	4022.04	3976.22	3956.08	3957.29
BIC	4042.09	4049.37	4025.41	4158.34	4165.00
Log Likelihood	-2006.11	-2006.02	-1979.11	-1941.04	-1940.64
ICC	0.59	0.59	0.61	0.56	0.56
Marginal R^2/Conditional R^2	0.000/0.588	0.000/0.588	0.018/0.616	0.136/0.620	0.137/0.621
Var: ID (Intercept)	4.70	4.70	5.11	4.19	4.20

$^{***}p < 0.001$; $^{**}p < 0.01$; $^{*}p < 0.05$

Table S18. Astronomical night: stratified analysis for self-reported sleep quality by IRBM.

Predictors	Null Model	Model 1	Model 2	Model 3	Model 4
Poor | Fair	-2.88***	-2.75***	-2.47***	-0.04	0.27
	(0.15)	(0.18)	(0.33)	(0.98)	(0.98)
Fair | Good	0.88***	1.00***	1.29***	3.73***	4.04***
	(0.12)	(0.16)	(0.32)	(0.99)	(0.99)
IRBM LAN		0.48	0.51	0.23	1.41
		(0.41)	(0.41)	(0.41)	(0.76)
Nap during the daytime: No			0.23	0.31	0.30
			(0.20)	(0.19)	(0.19)
Sleep day type: Weekday			0.14	0.15	0.15
			(0.11)	(0.11)	(0.11)
Wake-up day type: Weekday			0.13	0.14	0.14
			(0.13)	(0.13)	(0.13)
Daytime physical activity			-0.26	-0.13	-0.13
			(0.52)	(0.52)	(0.52)
Season: Summer				0.04	-0.02
				(0.52)	(0.51)
Season: Autumn				0.40	0.38
				(0.36)	(0.35)
Season: Winter				0.36	0.34
				(0.31)	(0.30)
Community: Central and Western				0.27	0.23
				(0.36)	(0.35)
Community: Kwun Tong				0.22	0.22
				(0.27)	(0.27)
Community: Kwai Tsing				-0.06	-0.04
				(0.31)	(0.30)
Overall health status. L				1.05***	1.05***
				(0.22)	(0.22)
Presence of preexisting sleep problems: No				0.47	0.48
				(0.37)	(0.36)
Sex: Female				0.22	0.62
				(0.23)	(0.32)
Age: 25-44				-0.36	-0.34
				(0.43)	(0.43)
Age: 45-65				-0.85	-0.82
				(0.52)	(0.51)
Marital Status: Others				-0.04	-0.02
				(0.52)	(0.51)
Marital Status: Married				0.38	0.37
				(0.30)	(0.29)
Education level. L				0.05	0.05

				(0.30)	(0.30)
Education level. Q				0.35	0.36
				(0.20)	(0.20)
Monthly household income. L				0.41	0.41
				(0.23)	(0.23)
Monthly household income. Q				-0.06	-0.04
				(0.18)	(0.18)
Employment status: Part-time				0.16	0.19
				(0.39)	(0.38)
Employment status: Freelance				1.01	1.07*
				(0.54)	(0.53)
Employment status: Student				0.21	0.20
				(0.43)	(0.42)
Employment status: Unemployed				-1.02**	-1.02**
				(0.38)	(0.37)
Household type: Couple without child				-0.06	-0.02
				(0.36)	(0.35)
Household type: Single parent				-0.06	-0.09
				(0.35)	(0.34)
Household type: Others				-0.46	-0.46
				(0.28)	(0.28)
Current smoker: No				0.46	0.43
				(0.58)	(0.57)
Current alcohol consumer: No				0.31	0.34
				(0.30)	(0.30)
Current coffee consumer: No				0.34	0.32
				(0.22)	(0.21)
Current energy drink consumer: No				0.58	0.60
				(0.40)	(0.39)
IRBM LAN: Sex Female					-1.59
					(0.89)
AIC	3042.70	3043.33	3046.47	3023.23	3033.21
BIC	3059.10	3065.19	3090.20	3220.02	3235.46
Log Likelihood	-1518.35	-1517.66	-1515.24	-1475.62	-1479.60
ICC	0.56	0.56	0.56	0.50	0.48
Marginal R^2/Conditional R^2	0.000/0.564	0.001/0.561	0.004/0.561	0.122/0.559	0.127/0.549
Var: ID (Intercept)	4.25	4.19	4.18	3.26	3.08

$^{***}p < 0.001$; $^{**}p < 0.01$; $^{*}p < 0.05$

Table S19. Astronomical night: stratified analysis for self-reported sleep quality by RMBM.

Predictors	Null Model	Model 1	Model 2	Model 3	Model 4
Poor | Fair	-2.88***	-2.82***	-2.56***	-0.08	0.06
	(0.15)	(0.20)	(0.33)	(0.98)	(1.00)
Fair | Good	0.88***	0.94***	1.21***	3.69***	3.83***
	(0.12)	(0.18)	(0.32)	(0.99)	(1.00)
RMBM LAN		0.17	0.22	0.08	0.56
		(0.37)	(0.37)	(0.37)	(0.64)
Nap during the daytime: No			0.23	0.31	0.30
			(0.20)	(0.19)	(0.19)
Sleep day type: Weekday			0.14	0.14	0.14
			(0.11)	(0.11)	(0.11)
Wake-up day type: Weekday			0.13	0.14	0.14
			(0.13)	(0.13)	(0.13)
Daytime physical activity			-0.36	-0.16	-0.19
			(0.53)	(0.53)	(0.53)
Season: Summer				0.04	0.03
				(0.52)	(0.52)
Season: Autumn				0.41	0.41
				(0.36)	(0.36)
Season: Winter				0.36	0.35
				(0.31)	(0.31)
Community: Central and Western				0.27	0.27
				(0.36)	(0.36)
Community: Kwun Tong				0.20	0.21
				(0.27)	(0.27)
Community: Kwai Tsing				-0.07	-0.07
				(0.31)	(0.31)
Overall health status. L				1.05***	1.04***
				(0.22)	(0.22)
Presence of preexisting sleep problems: No				0.46	0.47
				(0.37)	(0.37)
Sex: Female				0.23	0.49
				(0.23)	(0.37)
Age: 25-44				-0.36	-0.38
				(0.44)	(0.44)
Age: 45-65				-0.86	-0.87
				(0.52)	(0.52)
Marital Status: Others				-0.05	-0.04
				(0.52)	(0.52)
Marital Status: Married				0.37	0.37
				(0.30)	(0.30)
Education level. L				0.05	0.05

				(0.30)	(0.30)
Education level. Q				0.35	0.34
				(0.20)	(0.20)
Monthly household income. L				0.41	0.42
				(0.23)	(0.23)
Monthly household income. Q				-0.07	-0.06
				(0.18)	(0.18)
Employment status: Part-time				0.16	0.16
				(0.39)	(0.39)
Employment status: Freelance				1.01	1.02
				(0.54)	(0.54)
Employment status: Student				0.22	0.21
				(0.43)	(0.43)
Employment status: Unemployed				-1.01**	-1.01**
				(0.38)	(0.38)
Household type: Couple without child				-0.07	-0.07
				(0.36)	(0.36)
Household type: Single parent				-0.06	-0.07
				(0.35)	(0.35)
Household type: Others				-0.47	-0.47
				(0.29)	(0.29)
Current smoker: No				0.47	0.47
				(0.58)	(0.58)
Current alcohol consumer: No				0.31	0.30
				(0.30)	(0.30)
Current coffee consumer: No				0.35	0.34
				(0.22)	(0.22)
Current energy drink consumer: No				0.59	0.59
				(0.40)	(0.40)
RMBM LAN: Sex Female					-0.70
					(0.78)
AIC	3042.70	3044.50	3047.60	3023.50	3024.67
BIC	3059.10	3066.36	3091.33	3220.28	3226.92
Log Likelihood	-1518.35	-1518.25	-1515.80	-1475.75	-1475.34
ICC	0.56	0.56	0.56	0.50	0.50
Marginal R^2/Conditional R^2	0.000/0.564	0.000/0.564	0.003/0.563	0.122/0.560	0.122/0.560
Var: ID (Intercept)	4.25	4.25	4.23	3.27	3.27

$^{***}p < 0.001$; $^{**}p < 0.01$; $^{*}p < 0.05$

Table S20. Astronomical night: Impact of covariates removal on LAN-Sleep duration association (IRBM).

Covariates	Beta (Reduced)	% Change Beta	SE (Reduced)	% Change SE	P-value (Reduced)	% Change P-value	Level
Daytime physical activity	-0.42	3.86	0.22	-0.27	0.06	21.13	Night-Level
Wake-up day type	-0.45	1.74	0.22	-1.47	0.05	-1.24	Night-Level
Nap during the daytime	-0.44	0.33	0.22	-0.32	0.05	-0.03	Night-Level
Sleep day type	-0.44	0.07	0.22	0.13	0.05	-0.25	Night-Level
Age	-0.41	6.39	0.22	-0.16	0.06	35.23	Individual-Level
Community	-0.44	1.33	0.22	0.31	0.04	-7.55	Individual-Level
Education level	-0.43	2.42	0.22	-0.02	0.05	12.10	Individual-Level
Sex	-0.44	0.14	0.22	0.04	0.05	0.46	Individual-Level
Household type	-0.44	0.34	0.22	0.15	0.04	-2.30	Individual-Level
Monthly household income	-0.49	11.55	0.22	0.15	0.03	-43.93	Individual-Level
Employment status	-0.45	3.08	0.22	0.35	0.04	-15.24	Individual-Level
Marital status	-0.44	0.15	0.22	0.37	0.05	-1.04	Individual-Level
Overall health status	-0.44	0.40	0.22	0.13	0.05	1.29	Individual-Level
Season	-0.46	5.66	0.22	0.05	0.03	-24.12	Individual-Level

Current alcohol consumer	-0.44	0.26	0.22	0.04	0.05	-1.43	Individual-Level
Current coffee consumer	-0.43	3.16	0.22	0.25	0.05	14.62	Individual-Level
Current energy-drink consumer	-0.44	0.07	0.22	0.08	0.05	-0.73	Individual-Level
Current smoker	-0.43	1.38	0.22	0.06	0.05	6.41	Individual-Level
Presence of preexisting sleep problems	-0.46	4.32	0.22	0.02	0.04	-18.80	Individual-Level

Table S21. Astronomical night: Impact of covariates removal on LAN-Sleep efficiency association (IRBM).

Covariates	Beta (Reduced)	% Change Beta	SE (Reduced)	% Change SE	P-value (Reduced)	% Change P-value	Level
Daytime physical activity	-0.04	0.79	0.02	0.11	0.01	4.85	Night-Level
Wake-up day type	-0.04	0.48	0.02	-0.10	0.01	4.12	Night-Level
Nap during the daytime	-0.04	0.07	0.02	0.00	0.01	-0.51	Night-Level
Sleep day type	-0.05	3.25	0.02	0.09	0.01	-21.11	Night-Level
Age	-0.04	1.88	0.02	0.19	0.01	12.54	Individual-Level
Community	-0.04	4.45	0.02	0.26	0.02	33.56	Individual-Level
Education level	-0.04	0.76	0.02	0.09	0.01	4.77	Individual-Level
Sex	-0.04	0.43	0.02	-0.64	0.01	7.64	Individual-Level
Household type	-0.04	1.47	0.02	0.07	0.01	10.28	Individual-Level
Monthly household income	-0.04	5.07	0.02	0.36	0.02	38.39	Individual-Level
Employment status	-0.05	2.98	0.02	0.14	0.01	-19.89	Individual-Level
Marital status	-0.05	2.16	0.02	0.27	0.01	-15.79	Individual-Level
Overall health status	-0.04	1.81	0.02	0.03	0.01	13.19	Individual-Level
Season	-0.04	4.47	0.02	-0.27	0.02	38.35	Individual-Level
Current alcohol consumer	-0.04	0.46	0.02	0.02	0.01	3.12	Individual-Level

Current coffee consumer	-0.04	2.94	0.02	0.20	0.02	21.03	Individual-Level
Current energy-drink consumer	-0.04	1.28	0.02	-0.08	0.01	9.94	Individual-Level
Current smoker	-0.04	2.54	0.02	0.01	0.02	19.20	Individual-Level
Presence of preexisting sleep problems	-0.05	1.39	0.02	0.16	0.01	-10.38	Individual-Level

Table S22. Biological night: LAN exposure by IRBM and RMBM across geographic contexts.

**Name**	**Community**				**p value of Kruskal-Wallis rank sum test**
	**Sha Tin (Mean(SD))**	**Central and Western (Mean(SD))**	**Kwun Tong (Mean(SD))**	**Kwai Tsing (Mean(SD))**	
IRBM LAN	0.16(0.42)	0.10(0.30)	0.11(0.29)	0.18(0.45)	0.7915
RMBM LAN	0.07(0.24)	0.05(0.21)	0.07(0.24)	0.08(0.24)	0.4795
p value of Wilcoxon rank sum test	<0.001	0.006	<0.001	0.004	

Table S23. Biological night: LAN exposure by IRBM and RMBM across temporal contexts.

**Name**	**Sleep day type**		**p value of Mann-Whitney U test**	**Wake-up day type**		**p value of Mann-Whitney U test**
	**Weekend (Mean(SD))**	**Weekday (Mean(SD))**		**Weekend (Mean(SD))**	**Weekday (Mean(SD))**	
IRBM LAN	0.14(0.37)	0.15(0.39)	0.4522	0.15(0.39)	0.14(0.38)	0.3328
RMBM LAN	0.07(0.24)	0.07(0.23)	0.1232	0.07(0.25)	0.06(0.23)	0.1433
p value of Wilcoxon rank sum test	<0.001	<0.001		<0.001	<0.001	

Table S24. Biological night: Beta/Odds ratio in LAN exposure to sleep parameters by IRBM and RMBM (95% CI).

	Residence-based			Mobility-based			p-value of Wald tests
	Estimates/ Odds Ratios	CI	p-value	Estimates/ Odds Ratios	CI	p-value	
**Sleep duration**							
Model 1	0.43	(-0.04,0.91)	0.075	0.76*	(0.03,1.50)	0.042	
Model 2	0.38	(-0.09,0.85)	0.112	0.72*	(0.00,1.45)	0.050	
Model 3	0.45	(-0.01,0.91)	0.056	0.91*	(0.19,1.63)	0.013	
**WASO**							
Model 1	0.34*	(0.09,0.59)	0.007	1.47**	(1.10,1.85)	<0.001	<0.001
Model 2	0.32*	(0.08,0.57)	0.010	1.46**	(1.09,1.83)	<0.001	<0.001
Model 3	0.38**	(0.14,0.62)	0.002	1.48**	(1.11,1.85)	<0.001	<0.001
**Self-reported nighttime awakenings**							
Model 1	1.28	(0.56,2.94)	0.556	1.18	(0.33,4.20)	0.799	
Model 2	1.28	(0.55,2.98)	0.565	1.19	(0.33,4.29)	0.793	
Model 3	1.44	(0.63,3.32)	0.389	1.35	(0.38,4.86)	0.646	
**Self-reported sleep quality**							
Model 1	0.84	(0.35,2.01)	0.693	0.66	(0.18,2.39)	0.523	
Model 2	0.84	(0.35,2.01)	0.694	0.66	(0.18,2.41)	0.529	
Model 3	0.81	(0.34,1.91)	0.627	0.69	(0.19,2.48)	0.567	

Note. Model 1: LAN exposure only; Model 2: Model 1 + nap during the daytime, sleep day type, wake-up day type, daytime physical activity; Model 3: Model 2 + community, season, sex, age, marital status, education level, monthly household income, employment status, household type, overall health status, presence of preexisting sleep problems, current smoker, current alcohol consumer, current coffee consumer, current energy drink consumer. *=p<0.05;**=p<0.01.

Table S25. Biological night: stratified analysis for sleep duration by IRBM.

**Predictors**	**Null Model**	**Model 1**	**Model 2**	**Model 3**	**Model 4**
(Intercept)	6.56***	6.55***	6.88***	6.97***	6.95***
	(0.05)	(0.06)	(0.16)	(0.49)	(0.49)
IRBM LAN		0.43	0.38	0.45	1.28**
		(0.24)	(0.24)	(0.24)	(0.44)
Nap during the daytime: No			0.34**	0.34**	0.34**
			(0.11)	(0.11)	(0.11)
Sleep day type: Weekday			0.00	0.00	-0.00
			(0.07)	(0.07)	(0.07)
Wake-up day type: Weekday			-0.59***	-0.59***	-0.58***
			(0.07)	(0.07)	(0.07)
Daytime physical activity			-0.51	-0.46	-0.48
			(0.28)	(0.29)	(0.29)
Season: Summer				-0.51*	-0.50*

	(0.25)	(0.25)
Season: Autumn	-0.39*	-0.39*
	(0.18)	(0.17)
Season: Winter	-0.20	-0.20
	(0.15)	(0.15)
Community: Central and Western	0.22	0.23
	(0.17)	(0.17)
Community: Kwun Tong	0.12	0.12
	(0.13)	(0.13)
Community: Kwai Tsing	-0.10	-0.11
	(0.15)	(0.15)
Overall health status. L	-0.02	-0.02
	(0.11)	(0.11)
Presence of preexisting sleep problems: No	0.35*	0.35*
	(0.18)	(0.18)
Sex: Female	0.05	0.12
	(0.11)	(0.12)
Age: 25-44	-0.41	-0.41
	(0.21)	(0.21)
Age: 45-65	-0.67**	-0.67**
	(0.25)	(0.25)
Marital Status: Others	0.01	-0.00
	(0.25)	(0.25)
Marital Status: Married	0.03	0.01
	(0.15)	(0.14)
Education level. L	-0.04	-0.06
	(0.15)	(0.15)
Education level. Q	0.10	0.10
	(0.10)	(0.10)
Monthly household income. L	-0.28*	-0.28*
	(0.11)	(0.11)
Monthly household income. Q	0.15	0.15
	(0.09)	(0.09)
Employment status: Part-time	-0.01	-0.00
	(0.19)	(0.19)
Employment status: Freelance	-0.14	-0.13
	(0.26)	(0.26)
Employment status: Student	-0.17	-0.17
	(0.21)	(0.21)
Employment status: Unemployed	0.06	0.07
	(0.18)	(0.18)
Household type: Couple without child	0.12	0.13
	(0.17)	(0.17)
Household type: Single parent	-0.10	-0.10

				(0.17)	(0.17)
Household type: Others				-0.22	-0.21
				(0.14)	(0.14)
Current smoker: No				0.23	0.23
				(0.29)	(0.29)
Current alcohol consumer: No				0.09	0.10
				(0.15)	(0.15)
Current coffee consumer: No				0.10	0.09
				(0.10)	(0.10)
Current energy drink consumer: No				-0.02	-0.03
				(0.19)	(0.19)
IRBM LAN: Sex Female					-1.15*
					(0.52)
AIC	6435.95	6366.84	6300.54	6314.54	6311.66
BIC	6452.34	6388.68	6344.21	6511.06	6513.64
Log Likelihood	-3214.97	-3179.42	-3142.27	-3121.27	-3118.83
ICC	0.29	0.30	0.30	0.27	0.26
Marginal R^2/Conditional R^2	0.000/0.286	0.002/0.299	0.036/0.324	0.080/0.325	0.084/0.323
Var: ID (Intercept)	0.74	0.76	0.73	0.62	0.61
Var: Residual	1.83	1.79	1.72	1.72	1.72

$^{***}p < 0.001$; $^{**}p < 0.01$; $^{*}p < 0.05$

Table S26. Biological night: stratified analysis for sleep duration by RMBM.

Predictors	Null Model	Model 1	Model 2	Model 3	Model 4
(Intercept)	6.56***	6.55***	6.90***	6.98***	6.99***
	(0.05)	(0.05)	(0.16)	(0.49)	(0.49)
RMBM LAN		0.76*	0.72*	0.91*	0.73
		(0.38)	(0.37)	(0.37)	(0.57)
Nap during the daytime: No			0.34**	0.34**	0.34**
			(0.11)	(0.11)	(0.11)
Sleep day type: Weekday			0.01	0.01	0.01
			(0.07)	(0.07)	(0.07)
Wake-up day type: Weekday			-0.60***	-0.59***	-0.59***
			(0.07)	(0.07)	(0.07)
Daytime physical activity			-0.55	-0.52	-0.51
			(0.28)	(0.29)	(0.29)
Season: Summer				-0.53*	-0.53*
				(0.25)	(0.25)
Season: Autumn				-0.37*	-0.37*
				(0.18)	(0.18)

Season: Winter	-0.18	-0.18
	(0.15)	(0.15)
Community: Central and Western	0.22	0.22
	(0.17)	(0.17)
Community: Kwun Tong	0.12	0.12
	(0.13)	(0.13)
Community: Kwai Tsing	-0.11	-0.11
	(0.15)	(0.15)
Overall health status. L	-0.02	-0.01
	(0.11)	(0.11)
Presence of preexisting sleep problems: No	0.36*	0.36*
	(0.18)	(0.18)
Sex: Female	0.07	0.06
	(0.11)	(0.11)
Age: 25-44	-0.40	-0.40
	(0.21)	(0.21)
Age: 45-65	-0.68**	-0.68**
	(0.25)	(0.25)
Marital Status: Others	0.02	0.01
	(0.25)	(0.25)
Marital Status: Married	0.02	0.02
	(0.15)	(0.15)
Education level. L	-0.03	-0.03
	(0.15)	(0.15)
Education level. Q	0.15	0.15
	(0.10)	(0.10)
Monthly household income. L	-0.29**	-0.29**
	(0.11)	(0.11)
Monthly household income. Q	0.14	0.15
	(0.09)	(0.09)
Employment status: Part-time	0.00	0.00
	(0.19)	(0.19)
Employment status: Freelance	-0.13	-0.13
	(0.26)	(0.26)
Employment status: Student	-0.19	-0.19
	(0.21)	(0.21)
Employment status: Unemployed	0.08	0.08
	(0.18)	(0.18)
Household type: Couple without child	0.15	0.15
	(0.17)	(0.17)
Household type: Single parent	-0.13	-0.13
	(0.17)	(0.17)
Household type: Others	-0.23	-0.23
	(0.14)	(0.14)

Current smoker: No				0.28	0.27
				(0.29)	(0.29)
Current alcohol consumer: No				0.04	0.05
				(0.15)	(0.15)
Current coffee consumer: No				0.12	0.12
				(0.11)	(0.11)
Current energy drink consumer: No				-0.06	-0.06
				(0.19)	(0.19)
RMBM LAN: Sex Female					0.32
					(0.75)
AIC	6435.95	6364.31	6296.01	6308.55	6310.37
BIC	6452.34	6386.14	6339.68	6505.07	6512.35
Log Likelihood	-3214.97	-3178.15	-3140.00	-3118.28	-3118.19
ICC	0.29	0.30	0.30	0.27	0.27
Marginal R^2/Conditional R^2	0.000/0.286	0.002/0.301	0.038/0.326	0.082/0.327	0.082/0.327
Var: ID (Intercept)	0.74	0.76	0.73	0.62	0.62
Var: Residual	1.83	1.79	1.71	1.71	1.71

$^{***}p < 0.001$; $^{**}p < 0.01$; $^{*}p < 0.05$

Table S27. Biological night: stratified analysis for WASO by IRBM.

**Predictors**	**Null Model**	**Model 1**	**Model 2**	**Model 3**	**Model 4**
(Intercept)	1.19***	1.18***	1.26***	2.13***	2.12***
	(0.03)	(0.03)	(0.08)	(0.26)	(0.26)
IRBM LAN		0.34**	0.32**	0.38**	0.67**
		(0.13)	(0.13)	(0.12)	(0.23)
Nap during the daytime: No			0.13*	0.11	0.11
			(0.06)	(0.06)	(0.06)
Sleep day type: Weekday			-0.02	-0.02	-0.03
			(0.03)	(0.03)	(0.03)
Wake-up day type: Weekday			-0.18***	-0.18***	-0.18***
			(0.04)	(0.04)	(0.04)
Daytime physical activity			-0.14	-0.09	-0.09
			(0.15)	(0.15)	(0.15)
Season: Summer				-0.16	-0.16
				(0.14)	(0.14)
Season: Autumn				-0.27**	-0.27**
				(0.10)	(0.10)
Season: Winter				-0.25**	-0.24**
				(0.08)	(0.08)
Community: Central and Western				0.13	0.13
				(0.09)	(0.09)
Community: Kwun Tong				0.16*	0.16*
				(0.07)	(0.07)

Community: Kwai Tsing	-0.07	-0.07
	(0.08)	(0.08)
Overall health status. L	-0.07	-0.07
	(0.06)	(0.06)
Presence of preexisting sleep problems: No	-0.01	-0.01
	(0.10)	(0.10)
Sex: Female	-0.24***	-0.22***
	(0.06)	(0.06)
Age: 25-44	-0.15	-0.15
	(0.12)	(0.12)
Age: 45-65	-0.17	-0.17
	(0.14)	(0.14)
Marital Status: Others	0.08	0.08
	(0.14)	(0.14)
Marital Status: Married	-0.11	-0.12
	(0.08)	(0.08)
Education level. L	-0.04	-0.05
	(0.08)	(0.08)
Education level. Q	0.05	0.05
	(0.05)	(0.05)
Monthly household income. L	-0.18**	-0.18**
	(0.06)	(0.06)
Monthly household income. Q	0.06	0.07
	(0.05)	(0.05)
Employment status: Part-time	-0.10	-0.09
	(0.10)	(0.10)
Employment status: Freelance	-0.01	-0.01
	(0.14)	(0.14)
Employment status: Student	-0.06	-0.06
	(0.11)	(0.11)
Employment status: Unemployed	0.11	0.11
	(0.10)	(0.10)
Household type: Couple without child	0.08	0.09
	(0.09)	(0.09)
Household type: Single parent	-0.18*	-0.18*
	(0.09)	(0.09)
Household type: Others	-0.14	-0.14
	(0.08)	(0.08)
Current smoker: No	-0.28	-0.28
	(0.16)	(0.16)
Current alcohol consumer: No	0.10	0.10
	(0.08)	(0.08)
Current coffee consumer: No	0.03	0.03
	(0.06)	(0.06)

Current energy drink consumer: No				-0.06	-0.06
				(0.11)	(0.11)
IRBM LAN: Sex Female					-0.40
					(0.27)
AIC	4103.34	4061.78	4040.00	4028.61	4028.47
BIC	4119.74	4083.61	4083.67	4225.13	4230.44
Log Likelihood	-2048.67	-2026.89	-2012.00	-1978.31	-1977.23
ICC	0.35	0.36	0.37	0.32	0.32
Marginal R^2/Conditional R^2	0.000/0.346	0.005/0.366	0.017/0.378	0.091/0.379	0.092/0.379
Var: ID (Intercept)	0.24	0.26	0.26	0.20	0.20
Var: Residual	0.46	0.45	0.44	0.44	0.44

$^{***}p < 0.001$; $^{**}p < 0.01$; $^{*}p < 0.05$

Table S28. Biological night: stratified analysis for WASO by RMBM.

Predictors	Null Model	Model 1	Model 2	Model 3	Model 4
(Intercept)	1.19***	1.15***	1.26***	2.07***	2.07***
	(0.03)	(0.03)	(0.08)	(0.26)	(0.26)
RMBM LAN		1.47***	1.46***	1.48***	1.55***
		(0.19)	(0.19)	(0.19)	(0.29)
Nap during the daytime: No			0.13*	0.10	0.10
			(0.06)	(0.06)	(0.06)
Sleep day type: Weekday			-0.02	-0.02	-0.02
			(0.03)	(0.03)	(0.03)
Wake-up day type: Weekday			-0.18***	-0.18***	-0.18***
			(0.04)	(0.04)	(0.04)
Daytime physical activity			-0.18	-0.13	-0.13
			(0.15)	(0.15)	(0.15)
Season: Summer				-0.18	-0.18
				(0.13)	(0.13)
Season: Autumn				-0.26**	-0.26**
				(0.09)	(0.09)
Season: Winter				-0.22**	-0.22**
				(0.08)	(0.08)
Community: Central and Western				0.14	0.14
				(0.09)	(0.09)
Community: Kwun Tong				0.15*	0.15*
				(0.07)	(0.07)
Community: Kwai Tsing				-0.07	-0.07
				(0.08)	(0.08)
Overall health status. L				-0.07	-0.07
				(0.06)	(0.06)
Presence of preexisting sleep problems: No				-0.01	-0.01

				(0.09)	(0.09)
Sex: Female				-0.22***	-0.21***
				(0.06)	(0.06)
Age: 25-44				-0.16	-0.16
				(0.11)	(0.11)
Age: 45-65				-0.20	-0.20
				(0.14)	(0.14)
Marital Status: Others				0.08	0.08
				(0.13)	(0.13)
Marital Status: Married				-0.11	-0.11
				(0.08)	(0.08)
Education level. L				-0.02	-0.02
				(0.08)	(0.08)
Education level. Q				0.04	0.04
				(0.05)	(0.05)
Monthly household income. L				-0.20***	-0.20***
				(0.06)	(0.06)
Monthly household income. Q				0.07	0.07
				(0.05)	(0.05)
Employment status: Part-time				-0.07	-0.07
				(0.10)	(0.10)
Employment status: Freelance				-0.00	0.00
				(0.14)	(0.14)
Employment status: Student				-0.10	-0.10
				(0.11)	(0.11)
Employment status: Unemployed				0.15	0.14
				(0.10)	(0.10)
Household type: Couple without child				0.09	0.09
				(0.09)	(0.09)
Household type: Single parent				-0.21*	-0.21*
				(0.09)	(0.09)
Household type: Others				-0.16*	-0.16*
				(0.07)	(0.07)
Current smoker: No				-0.21	-0.21
				(0.15)	(0.15)
Current alcohol consumer: No				0.06	0.06
				(0.08)	(0.08)
Current coffee consumer: No				0.05	0.05
				(0.06)	(0.06)
Current energy drink consumer: No				-0.07	-0.07
				(0.10)	(0.10)
RMBM LAN: Sex Female					-0.12
					(0.38)
AIC	4103.34	4009.92	3987.34	3976.07	3977.96
BIC	4119.74	4031.76	4031.01	4172.58	4179.94

Log Likelihood	-2048.67	-2000.96	-1985.67	-1952.03	-1951.98
ICC	0.35	0.36	0.36	0.31	0.31
Marginal R^2/Conditional R^2	0.000/0.346	0.033/0.377	0.046/0.389	0.117/0.389	0.117/0.389
Var: ID (Intercept)	0.24	0.24	0.24	0.19	0.19
Var: Residual	0.46	0.44	0.43	0.43	0.43
$^{***}p < 0.001$; $^{**}p < 0.01$; $^{*}p < 0.05$					

Table S29. Biological night: stratified analysis for self-reported nighttime awakenings by IRBM.

Predictors	Null Model	Model 1	Model 2	Model 3	Model 4
0 | 1	-0.88***	-0.87***	-1.46***	-2.14*	-2.20*
	(0.12)	(0.13)	(0.30)	(1.05)	(1.05)
1 | 2	1.32***	1.33***	0.81**	0.14	0.08
	(0.13)	(0.13)	(0.30)	(1.05)	(1.05)
2 | ≥3	3.07***	3.09***	2.64***	1.96	1.91
	(0.15)	(0.16)	(0.31)	(1.05)	(1.05)
IRBM LAN		0.25	0.25	0.37	-1.18
		(0.42)	(0.43)	(0.43)	(0.84)
Nap during the daytime: No			-0.04	-0.07	-0.07
			(0.19)	(0.19)	(0.19)
Sleep day type: Weekday			-0.14	-0.14	-0.13
			(0.11)	(0.11)	(0.11)
Wake-up day type: Weekday			-0.81***	-0.82***	-0.82***
			(0.12)	(0.12)	(0.12)
Daytime physical activity			0.46	-0.03	-0.01
			(0.52)	(0.52)	(0.52)
Season: Summer				-1.27*	-1.29*
				(0.57)	(0.56)
Season: Autumn				-0.58	-0.58
				(0.39)	(0.39)
Season: Winter				-0.68*	-0.69*
				(0.33)	(0.33)
Community: Central and Western				-0.46	-0.47
				(0.39)	(0.39)
Community: Kwun Tong				0.48	0.48
				(0.30)	(0.29)
Community: Kwai Tsing				-0.01	-0.00
				(0.33)	(0.33)
Overall health status. L				-0.85***	-0.85***
				(0.24)	(0.24)
Presence of preexisting sleep problems: No				0.28	0.29
				(0.39)	(0.39)
Sex: Female				0.32	0.20

				(0.25)	(0.26)
Age: 25-44				0.71	0.72
				(0.48)	(0.48)
Age: 45-65				1.19*	1.19*
				(0.57)	(0.57)
Marital Status: Others				0.14	0.16
				(0.57)	(0.57)
Marital Status: Married				0.60	0.63*
				(0.32)	(0.32)
Education level. L				0.41	0.43
				(0.33)	(0.33)
Education level. Q				0.07	0.08
				(0.22)	(0.21)
Monthly household income. L				-0.32	-0.33
				(0.25)	(0.25)
Monthly household income. Q				0.10	0.10
				(0.20)	(0.20)
Employment status: Part-time				-0.02	-0.04
				(0.42)	(0.41)
Employment status: Freelance				-0.78	-0.81
				(0.57)	(0.57)
Employment status: Student				-0.34	-0.34
				(0.47)	(0.47)
Employment status: Unemployed				0.68	0.66
				(0.41)	(0.41)
Household type: Couple without child				0.84*	0.83*
				(0.38)	(0.38)
Household type: Single parent				0.14	0.14
				(0.38)	(0.38)
Household type: Others				0.28	0.28
				(0.31)	(0.31)
Current smoker: No				-0.56	-0.56
				(0.63)	(0.63)
Current alcohol consumer: No				-0.61	-0.62
				(0.33)	(0.32)
Current coffee consumer: No				0.13	0.13
				(0.23)	(0.23)
Current energy drink consumer: No				-0.18	-0.16
				(0.42)	(0.42)
IRBM LAN: Sex Female					2.10*
					(0.97)
AIC	4020.23	3992.71	3948.83	3929.13	3926.43
BIC	4042.09	4020.01	3997.96	4131.11	4133.87
Log Likelihood	-2006.11	-1991.36	-1965.42	-1927.57	-1925.22
ICC	0.59	0.59	0.61	0.56	0.56

Marginal R^2/Conditional R^2	0.000/0.588	0.005/0.592	0.018/0.617	0.136/0.621	0.140/0.620
Var: ID (Intercept)	4.70	4.77	5.15	4.22	4.17
$^{***}p < 0.001$; $^{**}p < 0.01$; $^{*}p < 0.05$					

Table S30. Biological night: stratified analysis for self-reported nighttime awakenings by RMBM.

Predictors	Null Model	Model 1	Model 2	Model 3	Model 4
0 | 1	-0.88***	-0.88***	-1.48***	-2.18*	-2.38*
	(0.12)	(0.13)	(0.30)	(1.06)	(1.07)
1 | 2	1.32***	1.31***	0.79**	0.10	-0.08
	(0.13)	(0.13)	(0.29)	(1.05)	(1.07)
2 | ≥3	3.07***	3.08***	2.61***	1.92	1.75
	(0.15)	(0.16)	(0.30)	(1.06)	(1.07)
RMBM LAN		0.16	0.17	0.30	-2.54*
		(0.65)	(0.66)	(0.65)	(1.07)
Nap during the daytime: No			-0.04	-0.07	-0.06
			(0.19)	(0.19)	(0.19)
Sleep day type: Weekday			-0.14	-0.13	-0.13
			(0.11)	(0.11)	(0.11)
Wake-up day type: Weekday			-0.81***	-0.82***	-0.84***
			(0.12)	(0.12)	(0.12)
Daytime physical activity			0.44	-0.07	-0.00
			(0.52)	(0.52)	(0.52)
Season: Summer				-1.27*	-1.34*
				(0.57)	(0.57)
Season: Autumn				-0.56	-0.55
				(0.39)	(0.39)
Season: Winter				-0.67*	-0.66*
				(0.33)	(0.33)
Community: Central and Western				-0.46	-0.48
				(0.39)	(0.39)
Community: Kwun Tong				0.49	0.49
				(0.30)	(0.30)
Community: Kwai Tsing				-0.01	-0.00
				(0.33)	(0.33)
Overall health status. L				-0.85***	-0.84***
				(0.24)	(0.24)
Presence of preexisting sleep problems: No				0.28	0.29
				(0.39)	(0.40)
Sex: Female				0.34	0.18
				(0.25)	(0.26)
Age: 25-44				0.71	0.72
				(0.48)	(0.49)

Age: 45-65				1.20*	1.18*
				(0.57)	(0.58)
Marital Status: Others				0.15	0.10
				(0.57)	(0.58)
Marital Status: Married				0.60	0.62
				(0.32)	(0.32)
Education level. L				0.41	0.43
				(0.33)	(0.33)
Education level. Q				0.07	0.07
				(0.22)	(0.22)
Monthly household income. L				-0.32	-0.34
				(0.25)	(0.25)
Monthly household income. Q				0.09	0.11
				(0.20)	(0.20)
Employment status: Part-time				-0.02	-0.02
				(0.42)	(0.42)
Employment status: Freelance				-0.77	-0.85
				(0.57)	(0.58)
Employment status: Student				-0.35	-0.38
				(0.47)	(0.48)
Employment status: Unemployed				0.68	0.71
				(0.41)	(0.41)
Household type: Couple without child				0.86*	0.86*
				(0.39)	(0.39)
Household type: Single parent				0.13	0.13
				(0.38)	(0.38)
Household type: Others				0.28	0.31
				(0.31)	(0.31)
Current smoker: No				-0.54	-0.72
				(0.63)	(0.64)
Current alcohol consumer: No				-0.64	-0.60
				(0.33)	(0.33)
Current coffee consumer: No				0.14	0.13
				(0.23)	(0.24)
Current energy drink consumer: No				-0.22	-0.19
				(0.43)	(0.43)
RMBM LAN: Sex Female					4.85***
					(1.38)
AIC	4020.23	3991.65	3947.46	3927.66	3916.74
BIC	4042.09	4018.94	3996.59	4129.63	4124.17
Log Likelihood	-2006.11	-2006.02	-1979.11	-1941.04	-1940.64
ICC	0.59	0.59	0.61	0.56	0.57
Marginal R^2/Conditional R^2	0.000/0.588	0.000/0.592	0.018/0.618	0.136/0.622	0.143/0.629
Var: ID (Intercept)	4.70	4.78	5.18	4.24	4.30

$^{***}p < 0.001$; $^{**}p < 0.01$; $^{*}p < 0.05$

Table S31. Biological night: stratified analysis for self-reported sleep quality by IRBM.

Predictors	Null Model	Model 1	Model 2	Model 3	Model 4
Poor | Fair	-2.88***	-2.89***	-2.68***	-0.17	-0.15
	(0.15)	(0.15)	(0.31)	(0.98)	(0.95)
Fair | Good	0.88***	0.87***	1.08***	3.59***	3.60***
	(0.12)	(0.12)	(0.30)	(0.99)	(0.96)
IRBM LAN		-0.18	-0.18	-0.21	1.37
		(0.44)	(0.45)	(0.44)	(0.86)
Nap during the daytime: No			0.20	0.27	0.28
			(0.20)	(0.20)	(0.20)
Sleep day type: Weekday			0.13	0.14	0.13
			(0.11)	(0.11)	(0.11)
Wake-up day type: Weekday			0.13	0.13	0.13
			(0.13)	(0.13)	(0.13)
Daytime physical activity			-0.34	-0.16	-0.21
			(0.53)	(0.53)	(0.52)
Season: Summer				0.11	0.12
				(0.52)	(0.51)
Season: Autumn				0.42	0.40
				(0.36)	(0.35)
Season: Winter				0.37	0.37
				(0.31)	(0.30)
Community: Central and Western				0.27	0.27
				(0.36)	(0.35)
Community: Kwun Tong				0.20	0.21
				(0.27)	(0.27)
Community: Kwai Tsing				-0.07	-0.08
				(0.31)	(0.30)
Overall health status. L				1.04***	1.03***
				(0.22)	(0.22)
Presence of preexisting sleep problems: No				0.47	0.45
				(0.37)	(0.36)
Sex: Female				0.23	0.35
				(0.23)	(0.23)
Age: 25-44				-0.37	-0.37
				(0.44)	(0.42)
Age: 45-65				-0.86	-0.86
				(0.52)	(0.51)
Marital Status: Others				-0.04	-0.06
				(0.52)	(0.51)
Marital Status: Married				0.36	0.34

				(0.30)	(0.29)
Education level. L				0.05	0.03
				(0.30)	(0.30)
Education level. Q				0.35	0.36
				(0.20)	(0.20)
Monthly household income. L				0.43	0.43
				(0.23)	(0.22)
Monthly household income. Q				-0.07	-0.06
				(0.18)	(0.18)
Employment status: Part-time				0.16	0.17
				(0.39)	(0.38)
Employment status: Freelance				1.06	1.08*
				(0.55)	(0.53)
Employment status: Student				0.23	0.22
				(0.43)	(0.41)
Employment status: Unemployed				-1.02**	-0.99**
				(0.38)	(0.37)
Household type: Couple without child				-0.08	-0.07
				(0.36)	(0.35)
Household type: Single parent				-0.07	-0.06
				(0.35)	(0.34)
Household type: Others				-0.49	-0.48
				(0.29)	(0.28)
Current smoker: No				0.47	0.46
				(0.58)	(0.57)
Current alcohol consumer: No				0.30	0.30
				(0.30)	(0.29)
Current coffee consumer: No				0.34	0.34
				(0.22)	(0.21)
Current energy drink consumer: No				0.58	0.56
				(0.40)	(0.39)
IRBM LAN: Sex Female					-2.15*
					(1.00)
AIC	3042.70	3022.67	3026.53	3002.33	3010.77
BIC	3059.10	3044.50	3070.20	3198.84	3212.75
Log Likelihood	-1518.35	-1507.33	-1505.26	-1465.16	-1468.39
ICC	0.56	0.56	0.56	0.50	0.48
Marginal R^2/Conditional R^2	0.000/0.564	0.000/0.563	0.002/0.562	0.121/0.559	0.128/0.545
Var: ID (Intercept)	4.25	4.23	4.21	3.27	3.02

$^{***}p < 0.001$; $^{**}p < 0.01$; $^{*}p < 0.05$

Table S32. Biological night: stratified analysis for self-reported sleep quality by RMBM.

Predictors	Null Model	Model 1	Model 2	Model 3	Model 4

Poor | Fair	-2.88***	-2.88***	-2.64***	-0.11	-0.02
	(0.15)	(0.15)	(0.31)	(0.98)	(0.98)
Fair | Good	0.88***	0.87***	1.11***	3.65***	3.75***
	(0.12)	(0.12)	(0.30)	(0.99)	(0.99)
RMBM LAN		-0.42	-0.42	-0.37	0.98
		(0.66)	(0.66)	(0.65)	(1.03)
Nap during the daytime: No			0.19	0.27	0.27
			(0.20)	(0.20)	(0.20)
Sleep day type: Weekday			0.13	0.13	0.13
			(0.11)	(0.11)	(0.11)
Wake-up day type: Weekday			0.14	0.14	0.15
			(0.13)	(0.13)	(0.13)
Daytime physical activity			-0.28	-0.09	-0.13
			(0.53)	(0.53)	(0.53)
Season: Summer				0.13	0.15
				(0.52)	(0.52)
Season: Autumn				0.39	0.39
				(0.36)	(0.36)
Season: Winter				0.37	0.36
				(0.31)	(0.31)
Community: Central and Western				0.27	0.28
				(0.36)	(0.36)
Community: Kwun Tong				0.18	0.19
				(0.27)	(0.27)
Community: Kwai Tsing				-0.07	-0.07
				(0.30)	(0.31)
Overall health status. L				1.04***	1.03***
				(0.22)	(0.22)
Presence of preexisting sleep problems: No				0.45	0.46
				(0.36)	(0.37)
Sex: Female				0.21	0.29
				(0.23)	(0.24)
Age: 25-44				-0.37	-0.37
				(0.43)	(0.43)
Age: 45-65				-0.87	-0.86
				(0.52)	(0.52)
Marital Status: Others				-0.04	-0.01
				(0.52)	(0.52)
Marital Status: Married				0.38	0.37
				(0.30)	(0.30)
Education level. L				0.05	0.04
				(0.30)	(0.30)
Education level. Q				0.36	0.36
				(0.20)	(0.20)

Monthly household income. L				0.43	0.44
				(0.23)	(0.23)
Monthly household income. Q				-0.06	-0.07
				(0.18)	(0.18)
Employment status: Part-time				0.16	0.16
				(0.38)	(0.39)
Employment status: Freelance				1.06	1.09*
				(0.54)	(0.55)
Employment status: Student				0.24	0.26
				(0.43)	(0.43)
Employment status: Unemployed				-1.02**	-1.03**
				(0.38)	(0.38)
Household type: Couple without child				-0.12	-0.11
				(0.36)	(0.36)
Household type: Single parent				-0.04	-0.04
				(0.35)	(0.35)
Household type: Others				-0.48	-0.49
				(0.28)	(0.29)
Current smoker: No				0.44	0.53
				(0.58)	(0.59)
Current alcohol consumer: No				0.34	0.32
				(0.30)	(0.30)
Current coffee consumer: No				0.32	0.33
				(0.22)	(0.22)
Current energy drink consumer: No				0.65	0.63
				(0.40)	(0.40)
RMBM LAN: Sex Female					-2.31
					(1.34)
AIC	3042.70	3023.63	3027.38	3002.75	3001.74
BIC	3059.10	3045.46	3071.05	3199.26	3203.72
Log Likelihood	-1518.35	-1507.81	-1505.69	-1465.37	-1463.87
ICC	0.56	0.56	0.56	0.50	0.50
Marginal R^2/Conditional R^2	0.000/0.564	0.000/0.561	0.002/0.561	0.122/0.557	0.124/0.560
Var: ID (Intercept)	4.25	4.20	4.18	3.24	3.25

$^{***}p < 0.001$; $^{**}p < 0.01$; $^{*}p < 0.05$

Code S1. Settings when processing the data with GGIR

```
GGIR(mode = c(1,2,3,4,5),
    datadir= "E:/Data/binband",
    outputdir= "E:/Data/GGIR_Sleep/Results_binband",
    studyname = c("BandSleep"),
    f0 = 1, f1 = 0,
    idloc = 6,
    print.filename = TRUE,
    #=====================
    # Part 1
    windowsizes = c(5,900,3600),
    do.enmo = TRUE,
    minloadcrit = 12,
    interpolationType = 1,
    printsummary = TRUE,
    #=====================
    # Part 2
    #=====================
    data_masking_strategy = 1,
    hrs.del.start = 0,        hrs.del.end = 0,
    maxdur = 0,               includedaycrit = 16,
    nonwear_approach = "2023",
    qwindow=c(0,24),
    mvpathreshold =c(100),
    #=====================
    # Part 3 + 4
    #=====================
    includenightcrit = 10,
    loglocation = "E:/Data/GGIR_Sleep/AdvancedSleeplog.csv",
    sleeplogsep = ",",
    sleepwindowType = "TimeInBed",
    outliers.only = FALSE,
    sleepefficiency.metric = 1,
    criterror = 4,
```

```
relyonguider = FALSE,
do.sibreport = TRUE,
do.visual = TRUE,
visualreport = TRUE,
#------------------------------
# Part 5 parameters:
#------------------------------
# Key functions: Merging physical activity with sleep analyses
threshold.lig = 30,
threshold.mod = 100,
threshold.vig = 400,
boutcriter = 0.8,
boutcriter.in = 0.9,
boutcriter.lig = 0.8,
boutcriter.mvpa = 0.8,
boutdur.in = c(10,20,30),
boutdur.lig = c(1,5,10),
boutdur.mvpa = c(1,5,10),
timewindow = c("WW"),
#----------------------------------
# Report generation
#------------------------------
do.report = c(2,4,5))
```

References

(1) Hees, V. T. van; Sabia, S.; Anderson, K. N.; Denton, S. J.; Oliver, J.; Catt, M.; Abell, J. G.; Kivimäki, M.; Trenell, M. I.; Singh-Manoux, A. A Novel, Open Access Method to Assess Sleep Duration Using a Wrist-Worn Accelerometer. *PLOS ONE* **2015**, *10* (11), e0142533. https://doi.org/10.1371/journal.pone.0142533.

(2) Sabia, S.; van Hees, V. T.; Shipley, M. J.; Trenell, M. I.; Hagger-Johnson, G.; Elbaz, A.; Kivimaki, M.; Singh-Manoux, A. Association Between Questionnaire- and Accelerometer-Assessed Physical Activity: The Role of Sociodemographic Factors. *Am. J. Epidemiol.* **2014**, *179* (6), 781–790. https://doi.org/10.1093/aje/kwt330.

(3) van Hees, V. T.; Gorzelniak, L.; Leon, E. C. D.; Eder, M.; Pias, M.; Taherian, S.; Ekelund, U.; Renström, F.; Franks, P.; Horsch, A.; Brage, S. Separating Movement and Gravity Components in an Acceleration Signal and Implications for the Assessment of Human Daily Physical Activity. *PLoS ONE* **2013**, *8* (4). https://doi.org/10.1371/journal.pone.0061691.

(4) Ding, L.; Chen, B.; Dai, Y.; Li, Y. A Meta-Analysis of the First-Night Effect in Healthy Individuals for the Full Age Spectrum. *Sleep Med.* **2022**, *89*, 159–165. https://doi.org/10.1016/j.sleep.2021.12.007.

(5) Tamaki, M.; Bang, J. W.; Watanabe, T.; Sasaki, Y. Night Watch in One Brain Hemisphere during Sleep Associated with the First-Night Effect in Humans. *Curr. Biol.* **2016**, *26* (9), 1190–1194. https://doi.org/10.1016/j.cub.2016.02.063.

(6) Bolker, B. M.; Brooks, M. E.; Clark, C. J.; Geange, S. W.; Poulsen, J. R.; Stevens, M. H. H.; White, J.-S. S. Generalized Linear Mixed Models: A Practical Guide for Ecology and Evolution. *Trends Ecol. Evol.* **2009**, *24* (3), 127–135. https://doi.org/10.1016/j.tree.2008.10.008.

(7) van Hees, V. T.; Fang, Z.; Langford, J.; Assah, F.; Mohammad, A.; da Silva, I. C. M.; Trenell, M. I.; White, T.; Wareham, N. J.; Brage, S. Autocalibration of Accelerometer Data for Free-Living Physical Activity Assessment Using Local Gravity and Temperature: An Evaluation on Four Continents. *J. Appl. Physiol.* **2014**, *117* (7), 738–744. https://doi.org/10.1152/japplphysiol.00421.2014.

(8) Craig, C. L.; Marshall, A. L.; Sjöström, M.; Bauman, A. E.; Booth, M. L.; Ainsworth, B. E.; Pratt, M.; Ekelund, U.; Yngve, A.; Sallis, J. F.; Oja, P. International Physical Activity Questionnaire: 12-Country Reliability and Validity. *Med. Sci. Sports Exerc.* **2003**, *35* (8), 1381–1395. https://doi.org/10.1249/01.MSS.0000078924.61453.FB.

(9) Harding, E. C.; Franks, N. P.; Wisden, W. The Temperature Dependence of Sleep. *Front. Neurosci.* **2019**, *13*, 336. https://doi.org/10.3389/fnins.2019.00336.

(10) Shi, D.; Dang, J.; Chen, H.; Yang, D.; Yu, Z.; Guo, L.; Dong, Y.; Li, J.; Li, X.; Li, X.; Li, X.; Song, Y. Assessment of Indoor Light-at-Night Exposure in Children and Adolescents during Schooldays and Weekends. *Environ. Pollut.* **2024**, *360*, 124689. https://doi.org/10.1016/j.envpol.2024.124689.

(11) Shi, D.; Li, J.; Dang, J.; Liu, Y.; Chen, Z.; Wang, Y.; Liu, J.; Wang, X.; Cai, S.; Zhang, Y.; Huang, T.; Chen, H.; Yang, D.; Yu, Z.; Guo, L.; Song, J.; Dong, Y.; Li, J.; Li, X.; Li, X.; Li, X.; Song, Y. Dual Associations of Post-Sleep and Pre-Wake Light-at-Night (LAN) Exposure with Myopia in Children and Adolescents. *Environ. Res.* **2025**, *279*, 121915. https://doi.org/10.1016/j.envres.2025.121915.

(12) Xu, Y.; Huang, Y.; Zhou, Y.; Wan, Y.; Su, P.; Tao, F.; Sun, Y. Association Between Bedroom Light Pollution With Subjectively and Objectively Measured Sleep Parameters Among Chinese Young Adults. *J. Adolesc. Health* **2024**, *74* (1), 169–176. https://doi.org/10.1016/j.jadohealth.2023.08.010.

(13) Obayashi, K.; Saeki, K.; Kurumatani, N. Association between Light Exposure at Night and Insomnia in the General Elderly Population: The HEIJO-KYO Cohort. *Chronobiol. Int.* **2014**, *31* (9), 976–982. https://doi.org/10.3109/07420528.2014.937491.

(14) Obayashi, K.; Saeki, K.; Iwamoto, J.; Ikada, Y.; Kurumatani, N. Association between Light Exposure at Night and Nighttime Blood Pressure in the Elderly Independent of Nocturnal Urinary Melatonin Excretion. *Chronobiol. Int.* **2014**, *31* (6), 779–786. https://doi.org/10.3109/07420528.2014.900501.

(15) Obayashi, K.; Saeki, K.; Kurumatani, N. Bedroom Light Exposure at Night and the Incidence of Depressive Symptoms: A Longitudinal Study of the HEIJO-KYO Cohort. *Am. J. Epidemiol.* **2018**, *187* (3), 427–434. https://doi.org/10.1093/aje/kwx290.

(16) Obayashi, K.; Tai, Y.; Yamagami, Y.; Saeki, K. Associations between Indoor Light Pollution and Unhealthy Outcomes in 2,947 Adults: Cross-Sectional Analysis in the HEIJO-KYO Cohort. *Environ. Res.* **2022**, *215*, 114350. https://doi.org/10.1016/j.envres.2022.114350.

(17) Obayashi, K.; Saeki, K.; Iwamoto, J.; Ikada, Y.; Kurumatani, N. Exposure to Light at Night and Risk of Depression in the Elderly. *J. Affect. Disord.* **2013**, *151* (1), 331–336. https://doi.org/10.1016/j.jad.2013.06.018.

(18) Obayashi, K.; Saeki, K.; Iwamoto, J.; Okamoto, N.; Tomioka, K.; Nezu, S.; Ikada, Y.; Kurumatani, N. Effect of Exposure to Evening Light on Sleep Initiation in the Elderly: A Longitudinal Analysis for Repeated Measurements in Home Settings. *Chronobiol. Int.* **2014**, *31* (4), 461–467. https://doi.org/10.3109/07420528.2013.840647.

(19) Esaki, Y.; Obayashi, K.; Saeki, K.; Fujita, K.; Iwata, N.; Kitajima, T. Effect of Nighttime Bedroom Light Exposure on Mood Episode Relapses in Bipolar Disorder. *Acta Psychiatr. Scand.* **2022**, *146* (1), 64–73. https://doi.org/10.1111/acps.13422.

(20) Esaki, Y.; Kitajima, T.; Obayashi, K.; Saeki, K.; Fujita, K.; Iwata, N. Light Exposure at Night and Sleep Quality in Bipolar Disorder: The APPLE Cohort Study. *J. Affect. Disord.* **2019**, *257*, 314–320. https://doi.org/10.1016/j.jad.2019.07.031.

(21) Johnson, D. A.; Wallace, D. A.; Ward, L. Racial/Ethnic and Sex Differences in the Association between Light at Night and Actigraphy-Measured Sleep Duration in Adults: NHANES 2011-2014. *Sleep Health* **2024**, *10* (1, Supplement), S184–S190. https://doi.org/10.1016/j.sleh.2023.09.011.

(22) Huss, A.; van Wel, L.; Bogaards, L.; Vrijkotte, T.; Wolf, L.; Hoek, G.; Vermeulen, R. Shedding Some Light in the Dark—A Comparison of Personal Measurements with Satellite-Based Estimates of Exposure to Light at Night among Children in the Netherlands. *Environ. Health Perspect.* **2019**, *127* (6), 067001. https://doi.org/10.1289/EHP3431.

(23) Mitsui, K.; Saeki, K.; Tone, N.; Suzuki, S.; Takamiya, S.; Tai, Y.; Yamagami, Y.; Obayashi, K. Short-Wavelength Light Exposure at Night and Sleep Disturbances Accompanied by Decreased Melatonin Secretion in Real-Life Settings: A Cross-Sectional Study of the HEIJO-KYO Cohort. *Sleep Med.* **2022**, *90*, 192–198. https://doi.org/10.1016/j.sleep.2022.01.023.

(24) Wallace-Guy, G. M.; Kripke, D. F.; Jean-Louis, G.; Langer, R. D.; Elliott, J. A.; Tuunainen, A. Evening Light Exposure: Implications for Sleep and Depression. *J. Am. Geriatr. Soc.* **2002**, *50* (4), 738–739. https://doi.org/10.1046/j.1532-5415.2002.50171.x.

(25) Beale, A. D.; Pedrazzoli, M.; Gonçalves, B. da S. B.; Beijamini, F.; Duarte, N. E.; Egan, K. J.; Knutson, K. L.; Schantz, M. von; Roden, L. C. Comparison between an African Town and a Neighbouring Village Shows Delayed, but Not Decreased, Sleep during the Early Stages of Urbanisation. *Sci. Rep.* **2017**, *7* (1), 5697. https://doi.org/10.1038/s41598-017-05712-3.

(26) Auger, R. R.; Burgess, H. J.; Dierkhising, R. A.; Sharma, R. G.; Slocumb, N. L. Light Exposure Among Adolescents With Delayed Sleep Phase Disorder: A Prospective Cohort Study. *Chronobiol. Int.* **2011**, *28* (10), 911–920. https://doi.org/10.3109/07420528.2011.619906.

(27) Khodasevich, D.; Tsui, S.; Keung, D.; Skene, D. J.; Revell, V.; Martinez, M. E. Characterizing the Modern Light Environment and Its Influence on Circadian Rhythms. *Proc. R. Soc. B Biol. Sci.* **2021**, *288* (1955), 20210721. https://doi.org/10.1098/rspb.2021.0721.
